# MicroRNA-driven periodontal immune homeostasis and tissue regeneration: from regulatory networks to engineering translation

**DOI:** 10.3389/fimmu.2026.1960089

**Published:** 2026-09-22

**Authors:** Xiaonan Wu, Yujia Wang, Yingjie Xu, Yingtao Wu

**Affiliations:** Qingdao Stomatological Hospital Affiliated to Qingdao University, Qingdao, Shandong, China

**Keywords:** biomaterial, extracellular vesicle, inflammation, microRNA (miRNA), osteogenic differentiation, periodontal immune homeostasis, periodontal regeneration

## Abstract

Periodontitis is a chronic immune-inflammatory disease induced by dental plaque biofilms. Persistent host immune dysregulation disrupts the dynamic balance between local tissue repair and pathological destruction, ultimately leading to irreversible damage to the periodontal supporting tissues, including the alveolar bone, periodontal ligament, and cementum. As key post-transcriptional regulators, microRNAs (miRNAs) coordinate the osteogenesis–bone resorption balance, immune homeostasis, angiogenesis, and intercellular communication through multi-target synergistic mechanisms, and have thus emerged as promising intervention targets for restoring periodontal immune homeostasis and achieving tissue regeneration. However, existing studies predominantly follow a simplified “single miRNA–single target–single phenotype” paradigm, and the three stages—molecular mechanism elucidation, delivery vector construction, and clinical validation—remain disconnected from one another. Published reviews likewise lack a comprehensive analytical framework that integrates multi-level miRNA regulatory networks with engineered delivery strategies. This review first systematically elucidates how endogenous microenvironmental factors, exogenous interventions, and mechanical stimuli regulate miRNA expression in periodontal tissues. It then examines the core functions of miRNAs across three dimensions—osteogenic/osteoclastic differentiation, immune homeostasis, and angiogenesis—analyzes their molecular interactions with competing endogenous RNAs (ceRNAs), summarizes the intercellular communication functions of miRNAs mediated by extracellular vesicles (EVs), and provides a graded synthesis of the research evidence for core miRNAs. At the translational engineering level, this review comprehensively surveys five strategies: miRNA mimic delivery, miRNA inhibition and blockade, indirect regulation of endogenous expression, engineered EV delivery, and DNA nanorobots. On this basis, it identifies three core bottlenecks constraining the field—the complexity of molecular regulatory networks, insufficient adaptability of delivery systems, and an incomplete preclinical validation framework—and proposes a spatiotemporally coordinated model for periodontal regeneration based on three sequential stages of intervention: “inflammation regulation, osteogenic–vascular coupling, and tissue maturation and remodeling.” This review aims to overcome the fragmentation of previous research by providing a systematic framework for elucidating the immune regulatory mechanisms of miRNA-mediated periodontal regeneration, developing smart responsive scaffolds, and conducting preclinical validation in large animals, while advancing the clinical translation of miRNA-based multi-target regulatory strategies in periodontal regeneration.

## Graphical Abstract

**Figure:**
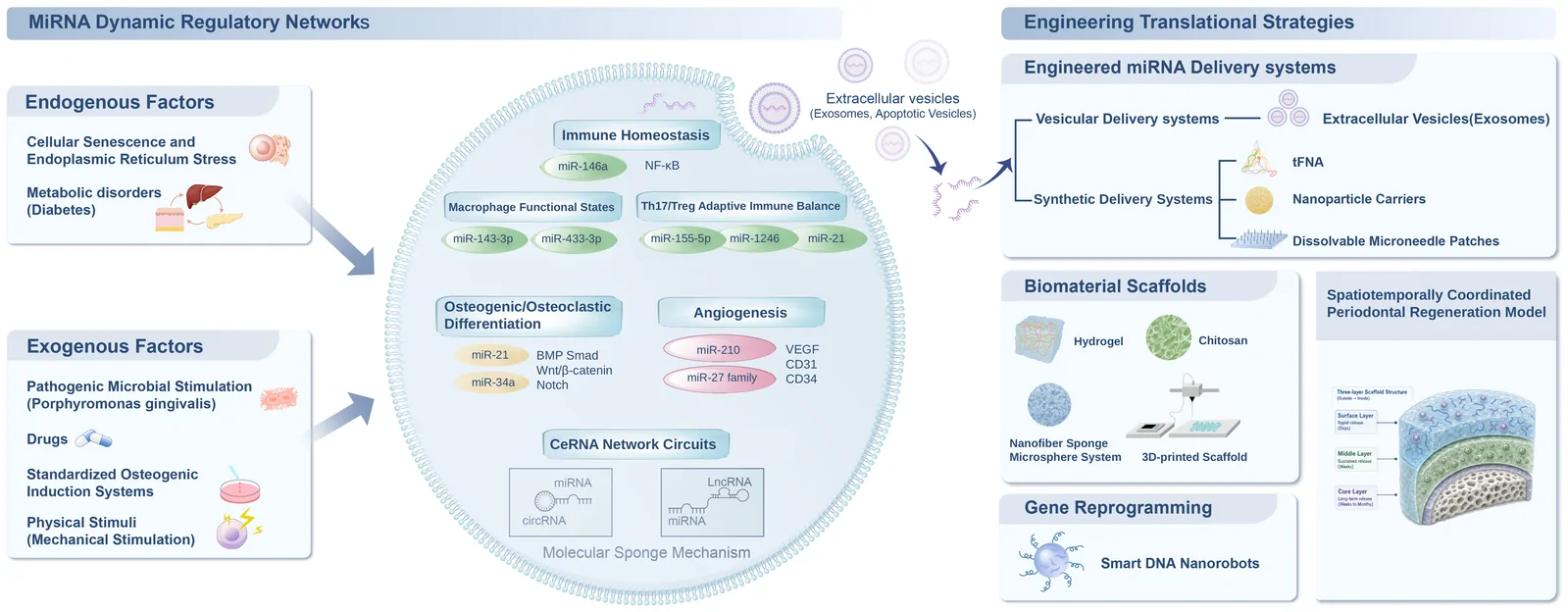

## Highlights

- Systematically integrates multi-level miRNA regulatory networks (osteogenesis/bone resorption, immune homeostasis, angiogenesis, competing endogenous RNA (ceRNA) interactions, and extracellular vesicle (EV) communication) and, based on cross-validation across multiple studies, provides evidence grading for core miRNAs, revealing the microenvironment-dependent mechanisms underlying their bidirectional functions.
- Comprehensively reviews five categories of engineered miRNA delivery strategies (miRNA mimic delivery, miRNA inhibition and blockade, indirect regulation of endogenous expression, engineered EV delivery, and DNA nanorobots) and cross-compares vesicular and synthetic delivery systems across seven dimensions.
- Analyzes the three major clinical-translation bottlenecks in miRNA-mediated periodontal regeneration—the complexity of molecular networks, insufficient adaptability of delivery systems, and an incomplete preclinical validation framework—and accordingly proposes a multi-omics analytical approach and a stepwise validation process spanning rodents, large animals, and clinical application.
- Proposes a novel spatiotemporally coordinated model for periodontal regeneration based on three sequential intervention stages (inflammation regulation, osteogenic–vascular coupling, and tissue maturation and remodeling), providing a theoretical framework for the design of smart responsive scaffolds.

## 1 Introduction

Periodontitis is a chronic immune-inflammatory disease induced by persistent stimulation from dental plaque biofilms. Its core pathological mechanism involves microbially induced dysregulation of host immune responses and the loss of local immune homeostasis, which perturbs the dynamic balance between physiological regeneration and pathological destruction of periodontal tissues and ultimately leads to irreversible structural damage to the periodontal supporting tissues, including the alveolar bone, periodontal ligament, and cementum. Of note, periodontitis exhibits well-established bidirectional associations with several systemic diseases, such as diabetes, cardiovascular disease, and rheumatoid arthritis, and severely compromises both oral function and systemic health (1). Although conventional periodontal debridement and guided tissue regeneration (GTR) can, to a certain extent, eliminate local pathogenic bacteria and control inflammatory progression, their capacity to repair and reconstruct severe periodontal defects remains limited because the persistently dysregulated inflammatory–immune microenvironment within the lesion continuously suppresses endogenous tissue repair signals, thereby hindering the restoration of periodontal tissue structure and function (2).

MicroRNAs (miRNAs) are a class of endogenous small non-coding RNAs that suppress gene expression primarily at the post-transcriptional level by binding complementarily to the 3′ untranslated region of target mRNAs (3). Unlike conventional growth factors or single-target drugs, miRNAs exert “one-to-many” and “pleiotropic” regulatory effects: a single miRNA can simultaneously regulate multiple downstream genes, finely coordinating repair processes such as inflammation resolution, angiogenesis, and bone remodeling across temporal and spatial dimensions (4, 5). In periodontal tissues, the regulatory roles of miRNAs span multiple dimensions. They can directly regulate alveolar bone metabolism by targeting key signaling pathways that govern osteogenesis and bone resorption (6–8); they can contribute to the maintenance of local immune homeostasis by modulating macrophage functional states and the balance of T-cell subsets (9, 10); and they can coordinate multicellular repair processes through extracellular vesicle (EV)–mediated intercellular communication (11). Notably, this multi-target regulatory mode of miRNAs is conceptually comparable to the dual-layer intervention paradigm—in which “multicomponent systemic regulation” coexists with “single-component precision targeting”—found in studies of natural products under the principle of “medicine–food homology” (12). Taking ginseng as an example, its dozens of active components, including ginsenosides, polysaccharides, and flavonoids, can systemically regulate immune cell function, inflammatory cytokine networks, and oxidative stress pathways (13). The monoterpene glycoside albiflorin, in turn, can exert anti-inflammatory and immunomodulatory effects across multiple physiological systems by targeting key nodes such as NF-κB and Toll-like receptor 4 (TLR4) (14). This dual-layer paradigm closely parallels the regulatory mechanisms of endogenous miRNA networks (multimolecular synergy) and individual miRNAs (single-molecule, multi-target action), providing a useful frame of reference for analyzing miRNA-mediated mechanisms of periodontal immune regeneration from a network perspective.

As research on miRNA regulatory mechanisms continues to advance, innovations in related technologies have become an important source of support. Bioinformatics and miRNA–mRNA regulatory network analyses provide systems-biology tools for elucidating the mechanisms underlying osteogenic differentiation of periodontal ligament stem cells (PDLSCs) (15) under inflammatory conditions (16). The combination of laser-capture microdissection and spatial omics technologies has made it possible to localize miRNA expression within specific tissue compartments (17), and the application of deep-learning algorithms to miRNA expression data has opened new avenues for biomarker screening and therapeutic-target prediction (18). These technological advances continue to drive research on miRNA regulatory mechanisms from single-molecule validation toward multi-omics integration.

Although numerous studies have confirmed the central regulatory role of miRNAs in periodontal regeneration, substantial integrative gaps remain in the current research framework. Most studies focus on a single miRNA or an isolated pathway and lack a systematic interpretation at the level of molecular interaction networks; existing reviews likewise tend to discuss molecular mechanisms or individual engineering techniques in isolation, without establishing a complete translational chain that connects regulatory mechanisms with delivery strategies. In light of these gaps, this review focuses on miRNA-mediated remodeling of periodontal immune homeostasis and tissue repair and regeneration. It systematically constructs a dynamic miRNA regulatory network, comprehensively summarizes engineering-based miRNA delivery strategies, analyzes the core bottlenecks and technical challenges constraining the field, and proposes a novel spatiotemporally coordinated model for periodontal regeneration, thereby establishing an integrated research framework that spans molecular regulation, smart delivery, and composite periodontal tissue regeneration. From an interdisciplinary perspective at the intersection of immunology and tissue engineering, this review establishes a comprehensive analytical framework that integrates fundamental mechanistic theory, bioengineering technology, and clinical translation, providing theoretical references and translational insights for the subsequent development of miRNA-based, targeted, and personalized treatment strategies for periodontal diseases.

(Note: This is a narrative review. A systematic search was conducted in the PubMed and Scopus databases (January 2015 to July 2026), combined with reference tracing and targeted manual searches to include some earlier classic literature and transferable studies from related fields. Details of the literature search and selection strategy are provided in the **Supplementary Materials**).

## 2 Regulatory mechanisms of miRNA expression in periodontal tissues

### 2.1 Endogenous regulation of miRNA expression in periodontal tissues

Because miRNA biogenesis entails a series of tightly regulated steps—including transcription, intranuclear cleavage, nuclear–cytoplasmic transport, and cytoplasmic processing (19)—alterations in the endogenous microenvironment can directly interfere with these processes and reshape the miRNA expression profile, thereby driving homeostatic disruption and pathological progression in periodontal tissues. In periodontal lesions, cellular senescence and endoplasmic reticulum stress induce disruption of competing endogenous RNA (ceRNA) regulatory networks and persistently impair the biological functions of periodontal cells (20). Metabolic disorders can remotely modulate periodontal miRNA expression through the circulation: for example, the abnormal expression of diabetes-associated miR-342-3p and miR-360, together with the effects of miR-221/222 and exosomal miR-124-3p under high-glucose conditions, disrupts periodontal cell function and accelerates periodontitis progression (21–23).

### 2.2 Exogenous regulation of miRNA expression in periodontal tissues

Exogenous interventions and environmental stimuli can reshape miRNA expression patterns and thereby contribute to the periodontal inflammatory response, cell differentiation, and tissue repair. Stimulation by pathogenic microorganisms is a key exogenous etiological factor: *Porphyromonas gingivalis* can activate the alternative polyadenylation (APA) mechanism (24), enabling macrophages to evade miRNA-mediated suppression, which induces uncontrolled local inflammation and disrupts microenvironmental homeostasis. Exogenous drugs—including psoralen (25), metformin (26), and betulinic acid (27)—as well as bioactive peptide–exosome combination regimens (28, 29) and standardized osteogenic induction systems (30), can all promote osteogenic differentiation of PDLSCs by regulating specific miRNA expression profiles or through targeted delivery mechanisms. In addition, environmental toxins and physical stimuli can markedly alter miRNA expression levels. Nicotine blocks the cell cycle and inhibits repair by upregulating miR-30a (31), whereas low-energy red-light LED irradiation downregulates miR-146a-5p and thereby enhances the osteogenic capacity of PDLSCs (32). Taken together, the miRNA expression profile is sensitive to exogenous stimuli; an in-depth analysis of the regulatory relationships between various interventions and miRNAs will provide new targets for the precision prevention and treatment of periodontal disease and for tissue regeneration.

### 2.3 Mechanical regulation of miRNA expression in periodontal tissues

The mechanical microenvironment can reshape the expression profiles of specific miRNAs and constitutes an important regulatory dimension through which periodontal tissues maintain homeostasis and respond to remodeling. Studies have confirmed that mechanical stimulation can reverse senescence in jawbone marrow mesenchymal stem cells and restore their osteogenic differentiation potential, with miR-210-3p and miR-214-3p showing altered expression in response to three-dimensional mechanical loading (33). Orthodontic tooth movement, a representative mechanical scenario of physiological remodeling in periodontal tissues, exhibits distinct miRNA expression patterns between the tension and compression zones: molecules such as miR-7a, miR-21-5p, and miR-100 show pronounced zone-specific distribution (34, 35), suggesting that mechanical loads can regulate miRNA expression along a spatial dimension. Furthermore, fluid shear stress upregulates miR-20a (36), and mechanical tension upregulates miR-21 (37), whereas miR-146a tends to be downregulated under compressive stimulation (38, 39). A systematic summary of additional mechanosensitive miRNAs and their regulatory mechanisms is provided in **Supplementary Table 1** (see **Supplementary Material**).

Notably, the interplay between mechanical stimulation and non-coding RNA networks can further amplify this regulatory effect. Wang et al. (2021) constructed a long non-coding RNA (lncRNA)–miRNA–mRNA network to elucidate the dynamic regulation of PDLSC fate by non-coding RNAs under mechanical stimulation (40) and Liu et al. (2024) demonstrated that mechanical stimulation promoted osteogenesis in PDLSCs via the circPRKD3/miR-6783-3p axis (41) providing a new molecular target for mechanically assisted periodontal regeneration.

## 3 Core regulatory functions of miRNAs in periodontal tissues and their interplay with ceRNA networks and extracellular vesicles

As key epigenetic molecules that maintain periodontal tissue homeostasis, miRNAs balance cell differentiation and regulate inflammatory responses by targeting genes and signaling pathways associated with osteogenesis, bone resorption, inflammation, and angiogenesis. Their regulatory scope is further broadened through interactions with ceRNA networks and EVs, forming a multilevel regulatory system of “molecular regulation—network synergy—intercellular communication” that provides a key molecular foundation and a set of intervention targets for periodontal regeneration.

### 3.1 miRNA-mediated regulation of osteogenic/osteoclastic differentiation in periodontal tissues

During physiological homeostasis and pathological repair of periodontal tissues, alveolar bone homeostasis depends on a dynamic, coupled balance between osteoblastic bone formation and osteoclastic bone resorption, in which miRNAs serve as key hubs regulating the relevant molecular pathways. To date, multiple miRNAs have been found to participate in fine-tuning these processes. For example, miR-508-5p directly targets and silences *SOX11*, thereby blocking osteogenic differentiation of PDLSCs (42), whereas miR-584-5p upregulates *H2AFZ*, which in turn downregulates the core transcription factor RUNX2 and inhibits the osteogenic potential of PDLSCs (43).

As research has progressed, however, evidence has shown that the same miRNA can exert opposing regulatory phenotypes in different microenvironments, with the bidirectional effects of miR-21 and miR-34a being the most representative examples. The bidirectional nature of miR-21 may arise from “dominant target selection” among distinct target pathways by microenvironmental signals. Under conventional osteogenic induction, the SMAD5 pathway predominates and miR-21 exerts an osteogenesis-inhibiting effect (44); by contrast, in a repair microenvironment enriched with growth factors, the PI3K/AKT/GSK3β/β-catenin axis is persistently activated and drives osteogenesis (45). Furthermore, miR-21 delivered by regulatory T cell (Treg) exosomes can promote osteogenic differentiation of PDLSCs through paracrine signaling (46), suggesting that the function of miR-21 may also depend on its mode of action; its immunoregulatory effects are discussed in detail in Section 3.2. miR-34a, in contrast, represents another bidirectional pattern—”one-to-many” functional antagonism among targets: it promotes osteogenesis by targeting *DKK1*, an antagonist of the Wnt pathway (47), while inhibiting osteogenesis by targeting *JAG1*, a Notch ligand (48). Its net effect may depend on the relative weight of each target pathway under a given cellular context, including cell type, differentiation stage, and the dominant signaling pathways in the microenvironment. Accordingly, seemingly contradictory findings in the literature should be interpreted by anchoring the analysis to contextual variables rather than by simply classifying a given miRNA as a pro-osteogenic or anti-osteogenic marker. Additional miRNAs involved in the regulation of osteogenesis and bone resorption, together with their detailed molecular mechanisms, are summarized in **Supplementary Table 2** (see **Supplementary Material**).

### 3.2 miRNA-mediated regulation of periodontal immune homeostasis

Beyond regulating osteogenic differentiation and periodontal bone metabolism, miRNAs are key epigenetic regulators that shape the local immune microenvironment and maintain periodontal immune homeostasis (49, 50). This homeostasis depends on a delicate balance among the cellular subsets of innate and adaptive immunity. Existing studies indicate that miRNAs participate in multiple processes—including macrophage functional-state transitions, the regulation of neutrophil apoptosis, and the T helper 17 (Th17)/Treg balance—and thus serve as important nodes in maintaining this equilibrium. Among the numerous miRNAs involved in periodontal immune regulation, miR-146a, a classic negative-feedback regulator of the Toll-like receptor (TLR)/IL-1R–NF-κB pathway, is induced by NF-κB and directly targets *IRAK1* and *TRAF6*, thereby inhibiting the secretion of pro-inflammatory cytokines such as TNF-α, IL-1β, and IL-6. This mechanism has been validated in human gingival fibroblasts (51), in the negative-feedback loop of periodontal ligament cells (PDLCs) stimulated with *Porphyromonas gingivalis* lipopolysaccharide (LPS) (52), and in PDLSCs (53), and it has shown an *in vivo* bone-protective effect in a collagen-induced arthritis model (54). Beyond such global inflammatory negative feedback, the fine regulation of specific immune cell subsets by miRNAs also profoundly shapes the homeostasis and trajectory of the periodontal immune network.

#### 3.2.1 miRNA regulation of macrophage functional states and osteoimmune interactions

As the core effector cells of innate immunity, macrophages constitute a highly heterogeneous immune-cell population with important regulatory roles in the periodontal microenvironment. *In vivo*, they do not adopt a fixed phenotype but form a highly plastic continuum of functional states (55), of which two are most representative. The pro-inflammatory state is characterized by secretion of TNF-α, IL-1β, and IL-6, expression of inducible nitric oxide synthase (iNOS), and high expression of CD86; its core functions include pathogen clearance, phagocytosis of necrotic tissue, and amplification of inflammatory signals. The reparative state is characterized by secretion of IL-10, TGF-β, and VEGF and by high expression of CD206 and CD163; its core functions include inflammation resolution, angiogenesis, extracellular matrix deposition, and clearance of apoptotic cells (efferocytosis). Of note, this functional-state continuum extends beyond these two poles: numerous intermediate transitional states exist between the pro-inflammatory and reparative states. Moreover, under pro-osteogenic microenvironmental cues—such as BMP/Wnt signaling and mechanical stimulation (56)—reparative-state macrophages can partially acquire a “regenerative” functional orientation, participating in new periodontal attachment formation and functional recovery.

Macrophage functional states are closely coupled with alveolar bone metabolism through osteoimmune interactions that largely influence the course of bone destruction and the regenerative potential in periodontitis. Specifically, pro-inflammatory macrophages secrete TNF-α, IL-1β, and other pro-inflammatory cytokines that upregulate local receptor activator of nuclear factor-κB ligand (RANKL) expression, elevate the RANKL/osteoprotegerin (OPG) ratio, promote osteoclast differentiation, and drive alveolar bone resorption. Reparative macrophages, by contrast, suppress inflammatory bone resorption through anti-inflammatory cytokines such as IL-10 and participate in osteogenic regulation through intercellular communication (57). Furthermore, a single-cell atlas of human periodontitis has revealed ligand–receptor communication between monocytes/macrophages and osteogenic-lineage cells in the osteoimmune microenvironment, and the Ephrin–Eph signaling axis has been implicated in bone regeneration after initial periodontal therapy (58) suggesting that macrophage–osteoblast interactions may constitute an important cellular basis for periodontal bone regeneration.

In periodontology, miRNAs act as key regulatory nodes in these osteoimmune interactions and exert bidirectional effects primarily through EVs. In the pro-inflammatory direction, PDLSCs in the inflammatory microenvironment can deliver pro-inflammatory miRNAs via exosomes/small extracellular vesicles (sEVs), driving macrophages toward a pro-inflammatory phenotype. Exosomes derived from inflammatory PDLSCs are enriched in miR-143-3p, which inhibits PI3K/AKT signaling by targeting PI3Kγ and activates NF-κB, driving pro-inflammatory skewing of macrophages and exacerbating periodontal inflammation *in vivo* (9). sEVs derived from LPS-pretreated PDLSCs target the TLR2/TLR4/NF-κB pathway via miR-433-3p and likewise induce pro-inflammatory features in macrophages (59). Notably, macrophage-derived EVs can also directly affect bone metabolism: apoptotic vesicles released by macrophages under inflammatory conditions are enriched in miR-143-3p and target *IGFBP5*, perturbing the osteogenesis–resorption balance and exacerbating periodontal bone resorption (60). In the reparative direction, exosomes overexpressing CXCR4 deliver miR-126 mimics specifically to macrophages at inflamed sites, promoting a shift toward a reparative phenotype and alleviating inflammation and bone loss in experimental periodontitis (61). A review of macrophage reprogramming also lists miR-126 as a candidate miRNA regulating reparative macrophage functions (62).

#### 3.2.2 miRNA regulation of neutrophil apoptosis and inflammation resolution

Neutrophils are the frontline immune defense in periodontitis; their excessive activation and delayed apoptosis are key mechanisms of sustained periodontal tissue injury. Conversely, timely neutrophil apoptosis and its effective clearance by macrophages (efferocytosis) are critical steps in inflammation resolution (50) However, direct evidence for the regulation of this process by miRNAs in the periodontal field is still lacking. Drawing on studies in other inflammatory models, hypoxia-induced apoptotic bodies from bone marrow mesenchymal stem cells (BMSCs) deliver miR-125b-5p to inhibit the PI3K/AKT pathway in neutrophils, thereby reversing LPS-induced delayed apoptosis and promoting neutrophil apoptosis and early healing in a mouse skin-wound model (63). These findings suggest that targeting miRNAs to regulate the timing of neutrophil apoptosis may be a promising strategy for promoting periodontal inflammation resolution, although its specific role in the periodontal microenvironment remains to be verified.

#### 3.2.3 miRNA regulation of adaptive immune responses via the Th17/Treg balance

The initiation of adaptive immune responses depends on antigen presentation by dendritic cells (DCs), but direct evidence that miRNAs regulate DC maturation and function in the periodontal field remains scarce. Current evidence focuses mainly on the effector phase of adaptive immunity, particularly the balance between T helper 17 (Th17) cells (which secrete IL-17 to recruit neutrophils and indirectly promote osteoclastogenesis) and regulatory T cells (Tregs) (which secrete IL-10/TGF-β to maintain immune homeostasis); multiple studies have confirmed that miRNAs play key roles in this balance.

At the cell-intrinsic level, IL-6 activation of the STAT3 pathway in BMSCs upregulates miR-181a-5p and mediates Th17/Treg imbalance, whereas pretreatment of BMSCs with a miR-181a-5p inhibitor attenuates this imbalance, promotes tissue regeneration, and reduces bone resorption in a mouse periodontitis model (64). At the intercellular communication level, miR-155-5p in PDLSC-derived exosomes regulates the Th17/Treg balance by targeting *SIRT1* (65), and BMSC-derived EVs suppress the target gene *ACE2* via miR-1246 and modulate YAP signaling, thereby reducing the Th17/Treg ratio in CD4+ T cells from patients with periodontitis and ameliorating experimental periodontitis (10).

Notably, the direction in which a given miRNA regulates the Th17/Treg balance is not fixed but may depend on its mode of action. miR-21 is a representative example. In the intercellular communication mode, Treg-derived exosomal miR-21 acts on PDLSCs through paracrine signaling, promoting their osteogenic differentiation and reducing periodontal defects (46); in the cell-intrinsic mode, by contrast, endogenous miR-21 in BMSCs inhibits TGF-β1 secretion through the PTEN/AKT/TGF-β1 axis, thereby suppressing Treg differentiation (66). This contrast indicates that the same miRNA can exert opposite effects on immune balance depending on whether it acts through intercellular communication or cell-intrinsic regulation. Moreover, miR-21 can influence osteoclast differentiation and orthodontic tooth movement by regulating the RANKL/OPG ratio in activated T cells (67).

At the translational level, nanofibrous sponge microspheres that differentially release miR-10a and IL-2/TGF-β locally recruit T cells and promote their differentiation into Tregs, achieving Treg enrichment and expansion in a mouse periodontitis model and thereby preventing alveolar bone loss (68). This strategy of co-delivering miRNAs and cytokines provides a feasible engineering approach for the precise regulation of adaptive immunity.

These advances, however, have focused primarily on miRNA regulation of T-cell responses. Although B cells and plasma cells infiltrate advanced periodontitis lesions extensively, direct evidence on miRNA regulation of B-cell activation and humoral immune responses remains limited. To date, only one study has reported that miR-146a regulates inflammatory cytokine secretion by B cells stimulated with *P. gingivalis* LPS by targeting *IRAK1* (rather than *TRAF6*) (69). Whether miRNAs participate in B-cell differentiation, antibody production, and broader humoral immune responses, and through what mechanisms, remain unclear and await further investigation.

#### 3.2.4 Integrative features and limitations of miRNA-mediated periodontal immune regulation

In summary, miRNA regulation of periodontal immune homeostasis does not rely on a single target or cell type but is distributed across multiple nodes—including macrophage functional-state regulation, the timing of neutrophil apoptosis, and the Th17/Treg balance—forming a distributed regulatory network. Notably, the functional bidirectionality highlighted above (for example, the opposing effects of miR-21 in cell-intrinsic versus intercellular communication modes) suggests that the immunoregulatory effects of miRNAs may be condition-dependent, with their net direction determined by the integration of cell source, mode of action, and microenvironmental signals. However, the current evidence remains based predominantly on *in vitro* cellular experiments and small-animal models, with little systematic validation across cell types and disease stages. In particular, notable gaps remain in DC antigen presentation and B-cell-mediated humoral immunity. These limitations also indicate that how miRNA-mediated immune regulation achieves spatiotemporal coupling with reparative processes such as osteogenic differentiation and angiogenesis still awaits elucidation through a more complete body of evidence. A more comprehensive summary of inflammation-related miRNAs and their regulatory pathways is provided in **Supplementary Table 3** (see **Supplementary Material**).

### 3.3 miRNA-mediated regulation of angiogenesis in periodontal tissues

#### 3.3.1 Functional characterization and research advances of key pro-angiogenic miRNAs

Periodontal tissues are highly vascularized, and their repair and regeneration depend heavily on timely reconstruction of the local vascular network. Angiogenesis not only supplies oxygen and nutrients to regenerating tissues but is also tightly coupled with osteogenic differentiation and inflammatory-microenvironment remodeling, constituting a key regulatory component of the periodontal regenerative microenvironment. Among the many angiogenesis-related miRNAs, evidence has concentrated on miR-126, miR-210, and the miR-27 family.

As a miRNA enriched in endothelial cells, miR-126 targets and represses *SPRED1* and *PIK3R2*, thereby relieving negative regulation of Ras/MAPK and PI3K/AKT signaling in vascular endothelial cells. This enhances cellular responsiveness to pro-angiogenic signals such as VEGF and promotes endothelial cell proliferation, migration, and tube formation (70). In periodontology, local targeted delivery of miR-126 has been shown to attenuate periodontal inflammation by inducing a reparative phenotype in macrophages (61) and to synergistically promote alveolar bone regeneration (71). However, direct evidence that miR-126 regulates angiogenesis within the periodontal microenvironment is still lacking. Given that its pro-angiogenic function has been thoroughly validated in vascular biology, miR-126 may serve as a bridge molecule linking periodontal vascular remodeling and immune homeostasis; its dual roles and mechanisms in periodontal regeneration warrant confirmation in future studies.

As a classic hypoxia-responsive miRNA (hypoxamir), miR-210 is upregulated in hypoxic microenvironments (72, 73) and contributes to angiogenesis by regulating hypoxic responses and tube formation through targeted repression of genes including ephrin-A3 in endothelial cells (73, 74). In periodontal tissue engineering, three-dimensional hydroxyapatite ceramic scaffolds upregulate miR-210 and promote VEGF secretion while inducing osteogenic differentiation of PDLSCs, suggesting that the miR-210/VEGF axis may contribute to the “vascularization–osteogenesis” coupling within the scaffold microenvironment (75) However, in-depth mechanistic validation of direct miR-210 targeting in the repair of *in situ* periodontal defects and of its crosstalk with the immune microenvironment is still lacking.

The miR-27 family displays a distinct osteogenic–angiogenic synergism. In vascular biology, miR-27b has been identified as a key regulator of vascular endothelial cell-fate determination; by repressing *Spry2* and *Dll4* (Notch ligands), it regulates endothelial tip-cell selection, sprouting, and venous specification, thereby fine-tuning angiogenesis (76). On the basis of this pro-angiogenic property, miR-27b mimics have shown considerable therapeutic potential in models of myocardial infarction and hindlimb ischemia—increasing vascular density in ischemic regions, improving tissue perfusion, and reducing fibrosis—thus offering a new strategy for therapeutic vascular regeneration (77). In osteogenic regulation, this family activates the Wnt/β-catenin pathway by repressing *SFRP1*, a classic Wnt antagonist, thereby promoting osteoblast differentiation (78). More recently, lipid nanoparticle delivery of miR-27 mimics was shown in a periodontitis model to upregulate osteogenic markers (ALP, RUNX2, COL1) and angiogenic factors (CD31, CD34, VEGF), induce a 6-fold increase in neovascularization, and shorten the alveolar crest–to–cementoenamel junction distance by 43.9%, thereby achieving functional periodontal regeneration (79).

Notably, marked functional heterogeneity exists among family members: miR-27a-5p attenuates periodontal inflammation by targeting *PTEN* (80), whereas in periodontitis, downregulation of the lncRNA *MEG3* relieves its sequestration of miR-27a-3p, increasing free miR-27a-3p, which in turn inhibits osteogenic differentiation of PDLSCs by targeting *IGF1* (81). Thus, members of the same family can exert opposing functional effects in inflammatory versus osteogenic microenvironments, indicating that the net effect of a given miRNA must be interpreted within its specific microenvironmental context.

#### 3.3.2 Signal convergence and cross-system synergy of pro-angiogenic miRNAs

As noted above, the regulatory effects of pro-angiogenic miRNAs are not confined to the vascular system; their downstream signaling nodes can simultaneously participate in multiple biological processes, including osteogenic differentiation and immune-microenvironment remodeling, thereby forming a synergistic network that spans cell lineages. Within this network, VEGF signaling is a central hub connecting distinct regulatory dimensions: miR-210 upregulates VEGF expression (75), and downstream VEGF signaling not only mediates endothelial cell proliferation and migration (70) but also induces mesenchymal stem cell differentiation toward the osteogenic lineage through the VEGFR/ERK/RUNX2 axis (82) and participates in macrophage polarization toward a reparative phenotype (83), thereby establishing the molecular basis for the “triple-axis coupling” of angiogenesis, osteogenic differentiation, and immune homeostasis. In parallel, the PI3K axis also shows cross-system signal convergence: miR-126 enhances PI3K signaling in endothelial cells by repressing *PIK3R2* (70), whereas in the osteogenic lineage the PI3K/β-catenin pathway is likewise a key module driving osteogenic differentiation of mesenchymal stem cells (45), suggesting that the same pathway can be differentially activated across cell types to cooperatively support overall periodontal regeneration.

Beyond cell-autonomous regulation by intracellular miRNAs, intercellular communication mediated by EVs constitutes another important layer of periodontal angiogenesis regulation. Under periodontitis conditions, EVs derived from dental pulp stem cells (DPSCs) deliver miR-378a to repress *SUFU* expression, thereby activating the Hedgehog/GLI1 pathway and promoting endothelial angiogenic capacity (84). This mechanism indicates that vascular remodeling in the periodontal microenvironment depends not only on reprogramming of the endothelial cells’ own miRNA expression profiles but also on paracrine, long-range regulation by neighboring stem cells via EVs.

In summary, pro-angiogenic miRNAs integrate angiogenesis, osteogenic differentiation, and immune homeostasis into a spatiotemporally synergistic network through multidirectional convergence of downstream signaling nodes and EV-mediated intercellular communication. Systematic elucidation of the cell-specific functions and microenvironment-dependent effects of individual miRNAs in this network will provide a theoretical basis and candidate targets for developing miRNA-based strategies for the coordinated regeneration of periodontal hard and soft tissues.

### 3.4 Regulatory mechanisms of ceRNA networks in periodontal tissues

Circular RNAs (circRNAs) and lncRNAs are important non-coding RNAs that competitively bind miRNAs through a “molecular sponge” mechanism, thereby relieving miRNA-mediated repression of downstream target genes and indirectly regulating periodontal cell differentiation and function (85–87).

In the circRNA network, circ_0054633 binds miR-590-3p to form a circ_0054633/miR-590-3p/*RUNX2* positive-feedback loop: RUNX2 promotes circ_0054633 transcription, and circ_0054633 acts as a molecular sponge to sequester miR-590-3p, thereby relieving this miRNA’s repression of *RUNX2*, sustaining *RUNX2* expression, and accelerating osteogenic differentiation of BMSCs (88). mmu_circ_0000217 acts as a ceRNA for miR-3064-3p, restoring expression of the downstream gene *Dkk1*, activating the JAK/STAT pathway, and promoting osteogenic differentiation of OCCM-30 cells (89).

In the lncRNA network, lncRNA *JHDM1D-AS1* competitively binds miR-532-5p, relieving its repression of *IGF1R*, activating the Notch/HES1 pathway, and ultimately promoting osteogenic differentiation of PDLCs (90). *MALAT1* can simultaneously bind miR-155-5p, miR-93-5p, and miR-124-3p to enhance osteogenesis through multiple pathways (91–93). *MEG8* binds miR-495-3p and miR-485-3p through the ceRNA mechanism, regulating *TCF4* expression and activating the Wnt/β-catenin pathway to maintain the osteogenic capacity of periodontal stem cells (94). Additional ceRNA networks and molecular pathways are comprehensively summarized in **Supplementary Table 4** (see **Supplementary Material**).

### 3.5 Mechanisms of extracellular vesicle-loaded miRNAs in regulating periodontal tissues

EVs—including exosomes, microvesicles, and apoptotic bodies—are central mediators of intercellular communication. They selectively encapsulate miRNAs and deliver them to recipient cells, participating in the balance between inflammation and regeneration, the maintenance of immune homeostasis, and the regulation of bone metabolism in periodontal tissues. The biological functions of EVs are strongly dependent on their cellular origin: vesicles secreted by different cell types carry markedly different functional cargo profiles and consequently exert distinct regulatory effects. Among EV subtypes, exosomes have been shown to act as natural miRNA carriers, transmitting functional genetic material between cells for long-range regulation (95, 96).

Vesicles derived from reparative cells (oral mesenchymal stem cells, reparative macrophages, and stem cells from exfoliated deciduous teeth) are enriched in osteogenic miRNAs that promote PDLSC differentiation (97–99). Exosomes secreted by periodontal ligament fibroblasts participate in local extracellular matrix remodeling through the miR-199a-5p/COL19A1 axis, thereby maintaining periodontal homeostasis (100). Apoptotic vesicles from mesenchymal stem cells deliver anti-osteoclastic miRNAs such as miR-223-3p, directly suppressing osteoclast activity and maintaining bone metabolic balance (101). Conversely, vesicles derived from pathological states—such as those from bacterially infected or inflamed macrophages—are typically enriched in pro-osteoclastic, anti-osteogenic miRNAs (miR-143-3p (60), miR-483-5p (102)) that induce alveolar bone resorption and inhibit osteogenic repair. Beyond bone metabolism, vesicle-loaded miRNAs are critical for inflammation resolution and immune regulation: apoptotic vesicles from hypoxia-induced BMSCs regulate neutrophil apoptosis via miR-125b-5p (63) (see Section 3.2.2), and platelet-rich fibrin-derived EVs achieve synergistic immune regulation and tissue regeneration through miR-21 and miR-146a (103).

**Supplementary Table 5** (see **Supplementary Material**) systematically summarizes “miRNAs mediating periodontal homeostasis via extracellular vesicles” across seven functional categories—osteogenesis promotion, osteogenesis inhibition, inflammatory and immune regulation, angiogenesis regulation, resorption regulation, dental-pulp repair, and physiological processes—consolidating the cellular sources, molecular mechanisms, and functional effects of key miRNAs to provide a comprehensive molecular basis for elucidating periodontal pathogenesis and developing targeted periodontal repair strategies.

### 3.6 Core miRNAs regulating periodontal immune homeostasis and tissue regeneration: multi-study evidence synthesis and functional analysis

Sections 3.1–3.5 have systematically organized the targeting relationships of various miRNAs in bone metabolism, immune inflammation, angiogenesis, ceRNA interactions, and EV-mediated signaling. However, most existing studies merely enumerate individual molecular pairings, lacking cross-comparisons and hierarchical analysis of experimental evidence. In this section, on the basis of the experimental systems and the extent of replicated validation in the literature, we selected core miRNAs with stable regulatory features and compiled them in **Table 1**, which compares their core signaling pathways, the scale of supporting literature, and the experimental systems used, so as to stratify the credibility of the evidence. The results show that miR-21 and miR-146a, supported by cross-validation from more than 10 independent studies, constitute the high-evidence-tier core of this table. In contrast, many miRNAs not included in the table are supported by only a few independent studies, lack functional validation in *in vivo* animal intervention models, or derive solely from high-throughput sequencing predictions; they are therefore insufficient, at present, to inform the design of targeted periodontal therapies. Even for miRNAs included in the table, such as miR-210 and miR-9-5p, the scale of independent supporting evidence remains relatively small (typically two to three studies), and they require replication across multiple laboratories and disease models together with corroborating *in vivo* functional experiments.

**Table 1:** Functional summary and evidence grading of core miRNAs regulating periodontal immune homeostasis and tissue regeneration.

No.	miRNA	Core function	Key target molecules/signaling pathways	Scope of independent supporting studies	Experimental support system
1	miR-21	Possesses dual potential to both promote and inhibit osteogenesis and is involved in the regulation of inflammation; its effects are microenvironment-dependent.	PI3K/AKT, BMP/SMAD, and PTEN-related pathways.	≥10 independent studies	*In vitro* and *in vivo* studies
2	miR-146a	Anti-inflammatory, maintains immune homeostasis; its effects on osteogenesis and resorption are microenvironment-dependent.	NF-κB and related inflammatory signaling pathways.	≥10 independent studies	*In vitro* and *in vivo* studies
3	miR-34a	Involved in osteogenesis and responses to mechanical stimulation; its effects are dependent on the weight of target pathways and the environment.	DKK1/Wnt, FGFR1, and JAG1/NOTCH.	≥4 independent studies	*In vitro* and *in vivo* studies
4	miR-143-3p	Promotes certain aspects of bone formation; this molecule in inflammation-derived EVs promotes inflammation and bone resorption in a source-dependent manner.	IGF2, IGFBP5, and PI3K/AKT/NF-κB.	≥3 independent studies	*In vitro* and *in vivo* studies
5	miR-155-5p	Involved in regulating Th17/Treg immune imbalance and osteogenic differentiation of PDLSCs.	SIRT1 and ETS1-related pathways.	≥3 independent studies	*In vitro* and *in vivo* studies
6	miR-181 family	Involved in inflammatory and immune dysregulation, osteoclast formation, and bone resorption; effects may vary among different family members.	IL-6/STAT3 and TNF-related pathways.	≥3 independent studies	*In vitro* and *in vivo* studies
7	miR-27 family	Promotes osteogenesis/dentinogenesis and angiogenesis, and may alleviate periodontal inflammation. (Specific members, such as miR-27a-3p, may inhibit osteogenesis.)	PTEN and Wnt-related pathways.	≥3 independent studies	*In vitro* and *in vivo* studies
8	miR-31-5p	Generally tends to inhibit osteogenesis and mineralization; the source of EVs may alter their periodontal effects.	Osterix, Satb2, and osteogenesis-related markers.	≥3 independent studies	*In vitro* and *in vivo* studies
9	miR-1246	Regulates immune homeostasis (restores Th17/Treg balance).	Nfat5/ACE2-related pathways.	≥2 independent studies	*In vitro* and *in vivo* studies
10	miR-126	Inhibits periodontal inflammation and promotes alveolar bone regeneration.	NF-κB/MAPK-related signaling pathways.	≥2 independent studies	*In vitro* and *in vivo* studies
11	miR-184	Generally tends to inhibit osteogenesis; inhibiting miR-184 may enhance osteogenesis.	NFI-C, PPARα, and Akt/JNK.	≥2 independent studies	*In vitro* and *in vivo* studies
12	miR-26a	Inhibits osteoclastogenesis; engineered exosome delivery promotes osteogenesis and bone repair.	RUNX1/JAG1 and exosome-mediated delivery-related pathways.	≥2 independent studies	*In vitro* and *in vivo* studies
13	miR-9-5p	Overall, it exhibits pro-inflammatory and osteoresorptive effects.	SIRT1/NF-κB-related pathways.	≥2 independent studies	*In vitro* and *in vivo* studies
14	miR-210	Responds to mechanical/material microenvironment and may promote the coupling of osteogenesis and angiogenesis.	VEGF-related pathways.	≥2 independent studies	*In vitro* studies

“Scope of independent supporting studies” encompasses direct periodontal studies as well as transferable studies in fields such as bone metabolism and immune regulation; “*in vitro* + *in vivo* studies” indicates that both cellular functional validation and animal intervention experiments have been completed. Further analysis of miRNA biological functions is provided in Sections 3.1–3.5 and **Supplementary Tables 1**-**5** in the **Supplementary Material**.

## 4 Engineered miRNA delivery strategies for periodontal regeneration

### 4.1 miRNA mimic delivery strategies

As a direct functional intervention, miRNA mimics replenish downregulated therapeutic miRNAs, particularly osteogenic and anti-inflammatory miRNAs whose expression is suppressed in pathological periodontal tissues. Chemical modifications such as 2′-O-methylation and locked nucleic acid (LNA) markedly enhance their nuclease resistance and serum stability (104). However, the clinical translation of mimics still faces substantial challenges. Because mimics possess a double-stranded RNA structure, they may activate innate immune responses through TLR3/7/8; moreover, their overexpression may compete with endogenous miRNAs for the intracellular small-RNA machinery, leading to reduced silencing efficacy of endogenous miRNAs and nonspecific changes in gene expression (105). In addition, mimics have a short *in vivo* half-life and often require repeated administration, and the relationship between dose and target-gene suppression is nonlinear; excessive doses may actually reduce specificity through saturation of the RNA-induced silencing complex (RISC). Rigorous dose–response curves must therefore be established to guard against immunogenic activation, off-target effects, and risks related to systemic biodistribution and organ safety.

In practical applications, various engineered delivery strategies have been developed to enhance the efficacy of mimics. For example, metal–polyphenol nanoparticles preserve the biological activity of miR-126 by promoting endosomal escape, achieving synergistic anti-inflammatory and microenvironment-remodeling effects in a periodontitis model (106). Lipid nanoparticles (LNPs) have been used for efficient encapsulation and delivery of miR-27 mimics, which synergistically promote osteogenic differentiation and angiogenesis by repressing *SFRP1* to activate the Wnt pathway (79). To address the challenge of local retention, dissolving microneedle patches co-delivering miR-126 nanoparticles and doxycycline effectively prolonged local retention and promoted alveolar bone regeneration (71). Tetrahedral framework nucleic acids (tFNAs), serving as multifunctional carriers for miR-200c, can target PDLSCs under inflammatory conditions; by regulating key genes of bone metabolism, they promote cell proliferation and osteogenic differentiation and thereby reduce bone loss in periodontitis models (107).

### 4.2 miRNA inhibition and blockade strategies

Complementary to miRNA mimic therapy, this strategy blocks osteogenesis-inhibiting or pro-inflammatory miRNAs that are upregulated under pathological conditions by delivering antisense oligonucleotides, LNA inhibitors, or miRNA sponges. Compared with mimics, inhibitors are typically single-stranded oligonucleotides that bind endogenous miRNAs through sequence-specific complementary pairing, with a relatively lower risk of off-target effects (108). However, their efficiency in suppressing high-abundance target miRNAs is constrained by intracellular concentration: sufficient levels must be reached to compete effectively for binding. Chemical modifications such as cholesterol conjugation can improve cellular uptake but may increase the risk of long-term toxicity (109). With respect to dose control, inhibitor dosage must be precisely matched to the endogenous abundance of the target miRNA; excessive use may cause nonspecific binding and global disruption of miRNA function. Long-term administration also requires vigilance regarding potential effects on liver and kidney function and the risks of complement activation and coagulation abnormalities.

For disease validation in periodontology, Wu et al. found that IL-6/STAT3 signaling in BMSCs mediates miR-181a-5p upregulation in experimental periodontitis; BMSCs pretreated with a miR-181a-5p inhibitor effectively attenuated Th17/Treg imbalance, promoted tissue regeneration, and reduced bone resorption in a ligature-induced murine periodontitis model (64), directly confirming the therapeutic potential of anti-miR strategies for periodontal immune imbalance. In terms of delivery technology, nanocarriers loaded with miR-146a-5p inhibitors can target endothelial cells to regulate angiogenesis; although this study was based on a skin-wound healing model, its targeted-delivery design offers an important reference for local periodontal application (110).

### 4.3 Indirect regulation of endogenous miRNA expression

Unlike the direct delivery of exogenous nucleic acids, this strategy uses biomaterials, small-molecule drugs, or physical stimuli to reshape the cellular endogenous miRNA expression profile, thereby achieving synergistic multi-target regulation. This approach can simultaneously regulate multiple miRNA networks and more closely approximates physiological regulation, and the biomaterials themselves often provide scaffold support (111). However, its mechanism is complex, its selectivity for individual miRNAs is limited, and its effects are strongly dependent on cellular state and microenvironment, making it difficult to precisely control the expression magnitude of any single miRNA. The relationship between the dose of a material or drug and the resulting changes in miRNA expression is nonlinear and is markedly influenced by cell type and culture conditions. Moreover, the safety of this strategy warrants careful attention: potential risks arise mainly from local microenvironmental toxicity of biomaterial degradation products and from systemic side effects associated with long-term use of small-molecule drugs, both of which require systematic *in vivo* validation (112).

Despite these challenges, the potential of this strategy to activate endogenous repair programs by reshaping the local microenvironment has been preliminarily demonstrated in periodontal tissue engineering. Existing studies indicate that three-dimensional hydroxyapatite scaffolds upregulate miR-210 and miR-129-5p, promoting vascularization–osteogenesis coupling and osteogenic differentiation, respectively (75, 113); a three-dimensional scaffold culture environment upregulates miR-2861 in human PDLSCs, accompanied by elevated RUNX2 protein levels and enhanced osteogenesis (114); and melatonin-loaded mesoporous wollastonite/chitosan scaffolds synergistically promote osteogenesis and angiogenesis while inhibiting osteoclast activity through the SERPINB9P1/miR-545-5p/SIRT6 axis, providing a strategy for balancing vascularization, osteogenesis, and osteoclastic activity in periodontal repair (115). In addition, small-molecule drugs such as psoralen (25) and metformin (26) have been shown to promote osteogenic differentiation of PDLSCs by regulating specific miRNA expression profiles (e.g., miR-125b-5p, miR-181a-5p).

### 4.4 Extracellular vesicle–mediated miRNA delivery strategies

#### 4.4.1 Bottlenecks and safety issues limiting EV-based miRNA delivery

Given the inherent potential of endogenous EVs for miRNA delivery, researchers have developed various engineering strategies to enhance their delivery performance. However, the translation of EVs from the laboratory to the clinic still faces multiple bottlenecks that require careful evaluation in the following key areas.

First, isolation and purification methods lack consensus and are prone to contamination by non-vesicular material. Although the MISEV2018 guidelines issued by the International Society for Extracellular Vesicles have established minimum requirements for EV isolation and characterization (116), methods such as ultracentrifugation, size-exclusion chromatography, and polymer precipitation rely on different physical principles, yielding vesicles that differ markedly in particle size, purity, and cargo profile. For example, ultracentrifugation and polymer precipitation are often accompanied by co-purification of non-vesicular material such as protein aggregates and lipoprotein complexes (117). These contaminants not only hamper cross-study comparison but may also independently mediate biological effects, leading to misinterpretation of EV-intrinsic functions (118).

Second, EV populations are highly heterogeneous. Even EVs secreted by the same cell type can form subpopulations—such as exosomes and microvesicles—through different biogenesis pathways, differing in particle size, density, surface markers, and miRNA cargo. Some studies still examine unfractionated, mixed EV populations, making it difficult to attribute specific functions precisely to particular subpopulations (119).

Third, biological effects are difficult to ascribe specifically to EV miRNAs. As composite delivery vehicles, EVs carry not only miRNAs but also mRNAs, proteins, and lipids; these components can mediate effects independently or synergistically. Most existing studies validate EV function indirectly by overexpressing or knocking down specific miRNAs in donor cells, whereas loss-of-function experiments in which specific miRNAs are directly removed at the EV level are relatively lacking. Consequently, the contribution of other cargo components to the observed phenotypes cannot be fully excluded.

Fourth, donor cells are heterogeneous and variable. Donor age, health status, passage number, and pretreatment conditions (e.g., hypoxia, inflammatory stimulation) all markedly affect EV secretion and cargo profiles. EVs derived from cell sources commonly used in periodontology—such as PDLSCs, BMSCs, and stem cells from human exfoliated deciduous teeth (SHEDs)—differ in potency, yet standardized cell-preparation and quality-control protocols are still lacking (120, 121).

Fifth, the reproducibility of exogenous cargo loading is inadequate. Loading methods such as electroporation, chemical transfection, and co-incubation differ substantially in encapsulation efficiency and cargo retention, with considerable batch-to-batch variation. Most studies report only single-batch loading efficiencies and lack systematic multi-batch evaluation, which is insufficient to support large-scale production (120, 122).

Sixth, dose definition and administration standards are not unified. Different studies use particle number, protein concentration, total RNA content, or donor-cell number as dose units, and these are not interconvertible; dose–response relationships for local periodontal administration have not been established, and no uniform standards exist for dosing frequency and intervals (123).

Seventh, *in vivo* biodistribution and local retention are poorly characterized. After systemic administration, EVs are readily cleared by the hepatosplenic mononuclear phagocyte system, and the fraction reaching the periodontium requires quantitative assessment. Local injection is subject to gingival crevicular fluid washout and masticatory forces, resulting in short retention and low bioavailability. Although scaffold-based delivery can prolong retention, data on release kinetics and diffusion range remain scarce (124, 125).

Eighth, target-cell uptake specificity is insufficient. EVs can enter cells through multiple uptake pathways—including membrane fusion, clathrin-mediated endocytosis, and caveolin-mediated endocytosis—that depend on cell type and surface molecular composition, and they lack adequate selective recognition of target cells such as PDLSCs and macrophages. Surface modification with targeting peptides or aptamers can improve specificity but may alter the native membrane-protein composition and immunological properties of EVs (126).

Ninth, storage stability is limited. During long-term storage at −80 °C, EVs may undergo aggregation, membrane fusion, and cargo degradation, and repeated freeze–thaw cycles can further reduce activity. Lyophilization can improve storage convenience but may alter vesicle membrane structure and targeting capacity. At present, room-temperature-stable formulations suitable for periodontal clinical settings are lacking (122).

Beyond these technical bottlenecks, EV clinical translation also faces serious safety challenges. For example, allogeneic EV components may trigger immune rejection, and long-term *in vivo* retention may lead to accumulation-related organ toxicity and potential tumor-promoting effects. Establishing comprehensive criteria for evaluating EV druggability and a full-lifecycle toxicological monitoring system is therefore a prerequisite for clinical application.

#### 4.4.2 Engineering strategies to overcome delivery bottlenecks for periodontal regeneration

To overcome these multiple technical barriers, targeted engineering of EVs and their donor cells is emerging as a key approach to breaking delivery bottlenecks and enhancing periodontal therapeutic efficacy. To improve loading efficiency and targeting, engineered sEVs enriched in miR-423-5p have been developed whose loading capacity is several orders of magnitude higher than that of native EVs and which can effectively induce early regeneration of the periodontal complex (cementum–periodontal ligament–alveolar bone) (127).

With respect to donor-cell engineering, exosomes secreted by P2X7 receptor–engineered stem cells carry miR-3679-5p, miR-6747-5p, and other miRNAs, targeting PDLSCs in the inflammatory microenvironment to reverse inflammation-induced osteogenic dysfunction (128). Transfection of BMSCs with the immunomodulatory peptide DP7-C also enables efficient loading of miR-26a into their secreted exosomes, promoting osteogenesis and attenuating bone damage in an experimental periodontitis model (53).

To improve formulation stability and local retention, a lyophilized exosome–hydrogel composite platform can prolong the local action of functional miRNAs such as let-7f-5p, enhancing osteogenic repair and reducing inflammation (129). EVs enriched in miR-5107-5p, secreted by erythropoietin (EPO)-pretreated macrophages and released locally from a chitosan hydrogel, restore the osteogenic function of BMSCs in an inflammatory environment through the EGFR/RhoA axis and markedly reduce alveolar bone resorption in a mouse periodontitis model (130). As cross-tissue corroboration, sEVs enriched in miR-451a and secreted by Mg²⁺-activated DPSCs, combined with β-tricalcium phosphate (β-TCP)-modified hydrogels, promote vascularized bone regeneration in a rat cranial defect model (131) offering an important reference for local sustained-release and vascular–osteogenic coupling designs in periodontology.

### 4.5 DNA nanorobots: miRNA-mediated cellular reprogramming

Beyond conventional delivery approaches, miRNA-mediated gene reprogramming offers a new strategy for the “targeted modification of diseased cells” in periodontitis. One study used smart DNA nanorobots integrating miR-1-3p with fibroblast-targeting peptides; after uptake by target cells in periodontal lesions, the nanorobots released the miRNA in response to intracellular glutathione, thereby repressing *FOSB* and *JUND* expression, reprogramming diseased-cell behavior, and promoting inflammation resolution and tissue regeneration (132). Although this strategy offers unique advantages in precise targeting, the application of DNA nanorobots in periodontology remains at the proof-of-concept stage, and a series of technical barriers must be overcome before clinical translation.

First, immunocompatibility. The immunogenicity of DNA nanostructures depends on their spatial conformation and the degree of CpG-motif exposure (133). Because the local immune microenvironment in periodontitis is already sensitized, nanorobot-triggered innate immune recognition could exacerbate local inflammation; the safety of both single and repeated administration therefore requires validation in appropriate models. Second, large-scale manufacturing. Although scale-up technologies are advancing, the mass production of clinical-grade DNA origami remains constrained by self-assembly efficiency, purification difficulty, batch consistency, and cost. Third, local stability and delivery efficiency. Because DNA structures are susceptible to nuclease degradation, further optimization and validation are needed to maintain structural integrity, prolong retention, and achieve effective tissue penetration and intracellular release in the complex periodontal microenvironment. Fourth, pharmacokinetics and long-term safety. No dedicated evaluation system exists for local periodontal administration; biodistribution, systemic exposure, and potential long-term off-target effects require systematic study.

In summary, these challenges indicate that DNA nanorobot-mediated gene reprogramming is unlikely to replace conventional delivery systems in the near term; nevertheless, its potential for precise targeting of diseased cells, stimuli-responsive controlled release, and synergistic multi-molecule loading should not be underestimated. If future breakthroughs are achieved in immunologically inert chemical modification, low-cost large-scale manufacturing, and periodontal-microenvironment adaptation, this technology may become an important cutting-edge adjunctive strategy for periodontal regeneration.

## 5 Discussion

### 5.1 Limitations and breakthrough pathways in current research

This review systematically integrates core miRNA targets, key signaling pathways, and EV interaction patterns to construct a multilevel, multidimensional dynamic miRNA regulatory network, and comprehensively surveys the latest advances in miRNA-related engineering technologies. Literature analysis indicates that although the reparative potential of miRNAs in periodontal regeneration has been preliminarily validated, most existing studies are confined to *in vitro* cellular experiments and small-animal models. Large-animal preclinical studies are scarce, and a substantial gap remains between basic mechanistic research and clinical translation. At its root, this gap does not arise from the absence of any single link but constitutes a systemic problem spanning the entire chain of “mechanism elucidation, vector delivery and clinical validation.”

#### 5.1.1 Complexity of miRNA regulatory networks and multi-omics solutions

Existing studies generally adopt a simplified reductionist paradigm of “single miRNA—single target—single phenotype,” which fragments complex, multilevel molecular regulatory networks. This makes it difficult to distinguish the differentiated immunoregulatory effects and temporal expression patterns of miRNAs across inflammatory stages, mechanical loads, and systemic metabolic conditions. At the study-design level, this limitation is reflected in an “isolated molecule—isolated phenotype” validation approach; at the technical level, it is reflected in reliance on one-dimensional miRNA expression profiles. The lack of multi-omics integration strategies may be one of the key technical reasons why the microenvironment-dependent functions of miRNAs have long remained unsystematically characterized and why conclusions regarding the same molecule (e.g., miR-21) are contradictory across studies. Moreover, conventional two-dimensional *in vitro* culture systems cannot recapitulate the dynamic pathological microenvironment of periodontal tissues, further aggravating the difficulty of reproducing *in vitro* positive findings *in vivo* and severely constraining the rational design of targeted intervention strategies.

Multi-omics integration strategies offer a viable route to overcoming these bottlenecks. Combined analysis of miRNA-seq and ATAC-seq in paired samples can correlate changes in miRNA expression with chromatin-accessibility dynamics, providing clues to the upstream epigenetic states and corresponding transcriptional outputs of candidate miRNAs; however, causal relationships still require validation through time-series and functional perturbation experiments (134). Spatial transcriptomics, combined with *in situ* miRNA detection or with miRNA profiling of adjacent sections, can resolve the cellular composition, gene expression programs, and miRNA-related regulatory contexts of distinct compartments—such as the sulcular epithelium, connective tissue, periodontal ligament, and alveolar bone—while preserving anatomical coordinates, thereby helping to explain functional differences of the same miRNA across microenvironmental contexts (135). Further integration with single-cell transcriptomics and chromatin-accessibility omics, combined with single-cell small-RNA profiling or spatial miRNA detection, can correlate cellular states, chromatin states, and transcriptomic outputs at higher resolution, providing a more refined technical approach to deciphering miRNA function. At present, however, studies employing such integrated strategies in periodontology remain scarce. Future work should promote the combined application of miRNA-seq, ATAC-seq, spatial transcriptomics, and single-cell multi-omics; through miRNA perturbation, target-gene rescue, and validation in independent cohorts, the spatiotemporal dynamics of miRNA regulatory networks in the periodontal microenvironment should be systematically dissected to bridge the gap between simplified research paradigms and real regulatory networks.

#### 5.1.2 Delivery system adaptability: challenges and strategies

At present, most mainstream delivery systems are static structures developed for general bone defects and have not been optimized for the unique periodontal microenvironment—namely, the high ribonuclease activity within the periodontal pocket, continuous washout by gingival crevicular fluid, and persistent inflammatory stimulation. In a high-nuclease environment, naked miRNAs are readily degraded; continuous washout by gingival crevicular fluid further shortens their local retention; and scaffolds lacking sequential-release capacity are prone to burst release. Together, these factors result in low local bioavailability of miRNAs at the lesion site and impede tissue regeneration. Moreover, even EVs, despite their favorable biocompatibility, are limited by the high cost of large-scale production and poor batch-to-batch consistency. Coupled with the absence of cross-comparative analyses and standardized optimization protocols across delivery vehicles, these issues make it difficult for researchers to select the optimal platform for a given periodontal application. Smart delivery carriers capable of responding to microenvironmental changes are therefore urgently needed to achieve precise, sequential administration of miRNAs.

To address the absence of such comparative analysis, this review provides a qualitative cross-comparison of vesicular and synthetic delivery systems across seven dimensions: loading efficiency, local retention and release kinetics, targeting specificity, immunogenicity, scalability, core advantages, and core limitations (**Table 2**). The two classes exhibit complementary structural strengths and weaknesses: vesicular systems offer relatively favorable biocompatibility and immune safety but face methodological and engineering bottlenecks (see Section 4.4), whereas synthetic systems have advantages in loading controllability and scalability but depend on surface modification for targeting, and some materials carry immunogenicity or toxicity risks. This comparison offers a reference for selecting delivery platforms according to specific periodontal therapeutic needs; future directions include the development of “vesicle–synthetic” hybrid delivery systems and the establishment of head-to-head quantitative comparison databases for the two platform classes.

**Table 2:** Multidimensional qualitative comparison of vesicular and synthetic miRNA delivery systems.

No.	Comparison dimensions	Vesicular delivery systems (extracellular vesicles/exosomes)	Synthetic delivery systems (LNPs, polymeric nanoparticles, metal–polyphenol nanoparticles, tetrahedral framework nucleic acids, dissolving microneedles, hydrogel scaffolds, DNA nanorobots, etc.)
1	Loading efficiency	Natural EVs have low and unstable drug-loading capacities; engineered loading methods (electroporation, transfection, co-incubation, etc.) can increase drug loading, but there are significant differences among these methods. Representative example: DP7-C-mediated loading of miR-26a was significantly higher than under control conditions.	Encapsulation efficiency can be regulated through formulation optimization, surface modification, and loading methods. Representative example: tetrahedral framework nucleic acids loaded with miR-200c via base pairing, resulting in high cellular uptake efficiency.
2	Local retention and release kinetics	Free EVs have a short local retention time and are easily flushed away by gingival crevicular fluid; however, their release can be prolonged when complexed with scaffold materials. Representative examples: miRNA achieves sustained release when complexed with β-TCP-modified GelMA; chitosan hydrogels loaded with EVs enable controlled release of miRNA.	Release kinetics can be regulated by adjusting the material degradation rate, crosslinking degree, and carrier structure. Representative examples: PLGA nanofiber microspheres achieved differentiated time-dependent release of miRNA and growth factors; dissolving microneedles prolonged local drug retention; and a chitosan/mesoporous wollastonite scaffold achieved local sustained release.
3	Targeting specificity	Natural EVs exhibit weak targeting and rely primarily on cell-derived tropism; surface modification can enhance specificity but may alter membrane protein composition and immunological properties. A representative example: P2X7R-genetically modified cell-derived exosomes can selectively rescue dysfunctional PDLSCs in the inflammatory microenvironment.	Cell-specific recognition can be achieved through surface-modified targeting ligands or peptides. Representative examples: YIGSR-peptide-modified nanocarriers targeting endothelial cells; DNA nanorobots achieving selective recognition of diseased cells via fibroblast-targeting peptides.
4	Immunogenicity	Immunogenicity is relatively low, and biocompatibility is generally good; however, differences in cell origin and non-vesicular contaminants may affect immunological safety.	Varies by material: biodegradable polymers such as chitosan exhibit low immunogenicity; LNPs may activate the complement system; the immunogenicity of DNA nanostructures depends on their spatial conformation and the degree of CpG sequence exposure.
5	Scalability	Large-scale production is challenging, with significant variations in particle count and protein concentration between batches. Upstream cell culture is influenced by donor age, passage number, and pretreatment conditions, and there is a lack of standardized GMP (Good Manufacturing Practice) processes	LNPs and polymeric nanoparticles are typically prepared using established processes such as microfluidics and emulsification, resulting in good batch-to-batch consistency; hydrogels and microneedles have established pathways for large-scale production; however, the clinical-scale mass production of DNA nanorobots remains limited by self-assembly efficiency and cost.
6	Core advantages	They exhibit good natural biocompatibility, relatively low immunogenicity, and can utilize membrane-mediated intracellular delivery, potentially with synergistic effects from endogenous molecules.	Good controllability of drug loading, structurally engineerable design, and scalable production pathways; localized drug delivery systems can prolong retention time.
7	Core limitations	Difficulties in large-scale production, significant batch-to-batch heterogeneity, unstable drug loading, weak targeting, and interference from non-vesicular contaminants.	Targeting depends on surface modification; some materials pose immunogenicity/toxicity risks; and there is a lack of synergistic effects with endogenous molecules.

1. This table compares the core characteristics of vesicular delivery systems (EVs/exosomes) and synthetic systems (lipid nanoparticles, polymeric nanoparticles, metal–polyphenol nanoparticles, tetrahedral framework nucleic acids, dissolving microneedles, hydrogel scaffolds, and DNA nanorobots) across seven dimensions.

2. Because the field currently lacks standardized studies performing head-to-head engineering characterization of the two classes under uniform experimental conditions, quantitative outcomes—such as encapsulation efficiency, release kinetics, and targeting efficiency—vary markedly across studies owing to differences in cell type, animal model, administration route, and preparation method, and cannot be generalized into universal quantitative benchmarks. This table therefore adopts a primarily qualitative descriptive format.

#### 5.1.3 Clinical translation barriers and preclinical validation

At present, many studies still use relatively simple mouse or rat bone-defect models and fail to adequately incorporate clinically relevant comorbidities such as diabetes and plaque infection; standardized animal strains, defect specifications, and modeling methods are also lacking across studies. Treatment evaluation relies solely on short-term micro-CT measurements of bone volume, which may overlook the layered functional attachment of alveolar bone, periodontal ligament, and cementum—a critical criterion for periodontal regeneration. Furthermore, systematic evaluation of long-term off-target effects, *in vivo* biodistribution, and immunotoxicity after repeated miRNA administration remains lacking, resulting in an incomplete safety assessment framework and increasing uncertainty in the clinical translation of miRNA therapies. To address these bottlenecks, this review proposes a stepwise preclinical validation framework that progresses from rodents to large animals and from mechanistic validation to safety assessment.

Phase I (rodent mechanistic validation): Candidate-miRNA screening, preliminary efficacy validation of delivery systems, and short-term safety assessment are completed in mouse/rat models of periodontitis or bone defects. Comorbidity models such as diabetes and plaque infection should be incorporated as needed to enhance clinical relevance.

Phase II (large-animal translational validation): This phase serves as an essential bridge between basic research and clinical trials; careful selection among three model types—dogs, minipigs, and non-human primates—is recommended according to the research objectives. Dogs (represented by Beagles) are the most established large-animal model in periodontal research (136): their periodontal anatomy and dentition resemble those of humans, their oral dimensions are manageable, and ligation-induced modeling methods are mature and reliable with good experimental tractability, making them suitable for delivery-system kinetics and mid-term safety assessment. Minipigs (e.g., the Minnesota minipig) closely resemble humans in craniofacial anatomy, histopathophysiology, and disease characteristics and can spontaneously develop gingivitis and periodontitis (137, 138); they are suitable for translational evaluation of complex periodontal defect models and guided tissue regeneration strategies but require demanding perioperative care. Non-human primates (e.g., rhesus macaques and cynomolgus monkeys) are highly consistent with humans in immune responses, inflammatory regulation, and bone metabolism and can naturally develop periodontitis (138); they are indispensable for efficacy prediction and long-term safety assessment of miRNA-based immunomodulatory therapies but, owing to strict ethical review and high costs, are generally reserved for pivotal end-point validation. In the large-animal phase, three aspects should be prioritized: (i) distribution, retention, and release kinetics of the delivery system in large-animal periodontal defects; (ii) stratified regenerative outcomes after treatment, specifically the synchronous regeneration of alveolar bone, periodontal ligament, and cementum assessed by histology, immunohistochemistry, and micro-CT; and (iii) immune safety and off-target effects over an extended observation period, including systemic immune-marker monitoring, histopathological examination of vital organs, and *in vivo* miRNA biodistribution analysis.

Phase III (preparation for clinical translation): On the basis of large-animal validation data, good manufacturing practice (GMP)-grade carrier preparation processes and quality-control standards should be established, an appropriately designed randomized controlled clinical trial protocol should be developed, and a personalized miRNA target-screening platform should be constructed.

By introducing large-animal models, this stepwise validation framework provides a practical roadmap for bridging the gap between rodent research and clinical translation. Nevertheless, it remains a theoretical framework at present. Large-animal models entail high ethical and temporal costs, and issues such as standardization of periodontal defects and harmonization of evaluation metrics still require field consensus; the effectiveness of each stage awaits verification and refinement through future research and practice.

### 5.2 A theoretical spatiotemporally coordinated model for periodontal regeneration

Building on recent advances in multilevel miRNA regulatory networks and delivery systems, and centering on the restoration of local immune homeostasis, this review follows the natural immune-repair sequence of “first controlling chronic inflammation, then initiating tissue regeneration” to propose a spatiotemporally coordinated model for periodontal regeneration, characterized by targeted recognition, sequential sustained release, multi-network synergistic regulation, and synchronous regeneration of hard and soft tissues. Because research in this area remains at an exploratory stage, this model is primarily a forward-looking theoretical framework derived through integration and extrapolation of existing evidence; systematic experimental validation still awaits further refinement and confirmation through future *in vitro* and *in vivo* studies. The overall architecture and three-phase design of this spatiotemporal-synergistic model are schematically illustrated in **Figure 1**.

**Figure 1:**
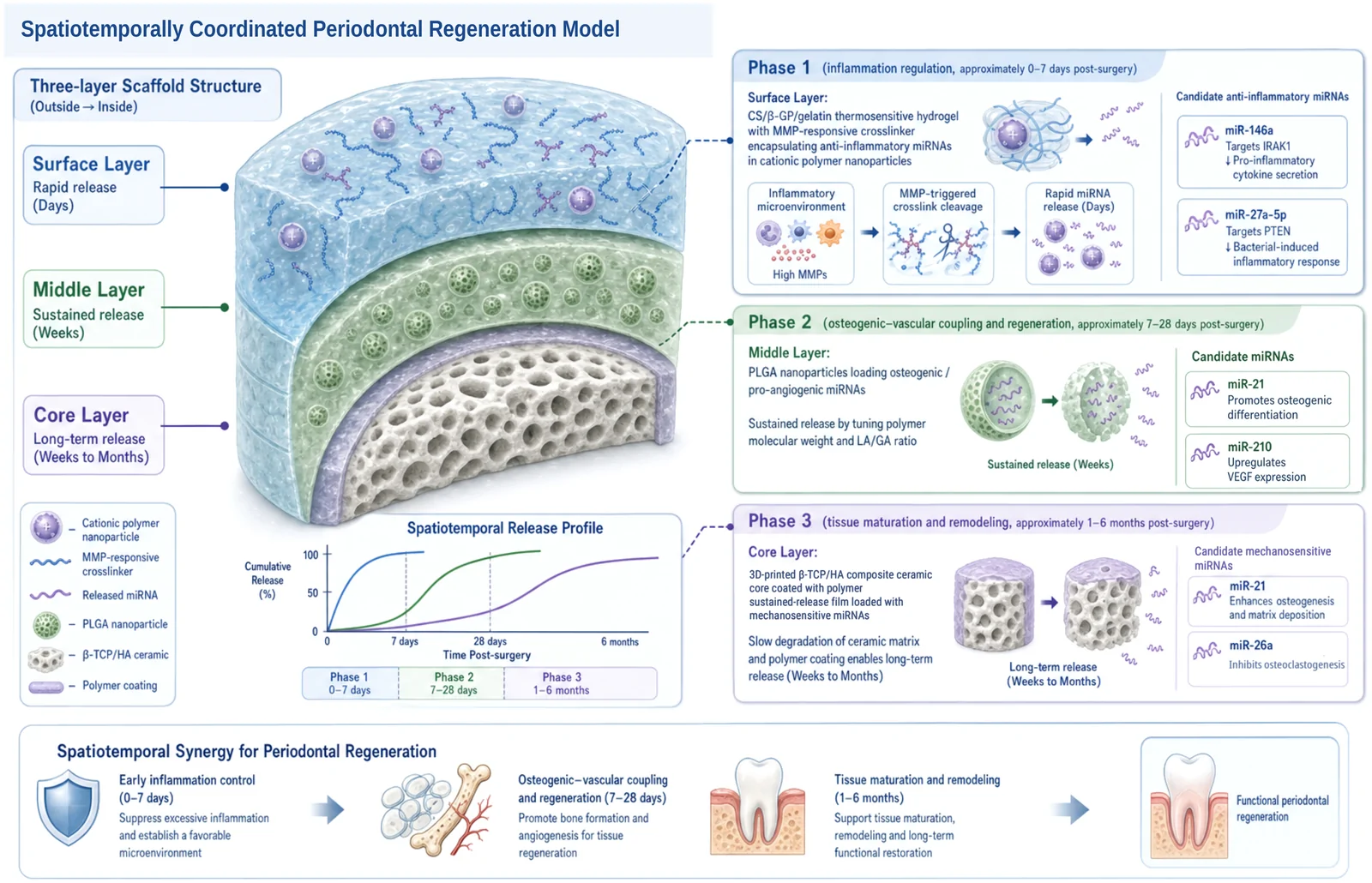
A theoretical spatiotemporally coordinated model for periodontal regeneration. The scaffold comprises a rapidly degradable surface layer (chitosan/β-glycerophosphate/gelatin thermosensitive hydrogel), a sustained-release middle layer (PLGA nanocarriers), and a long-term core layer (3D-printed β-TCP/HA ceramic), whose sequential degradation drives three temporally coordinated repair phases: inflammation regulation (0–7 days), osteogenic–vascular coupling and regeneration (7–28 days), and tissue maturation and remodeling (1–6 months), each associated with distinct candidate miRNAs and dominant molecular pathways.

To clarify the core logic and translatable design of this model, we present a conceptual, illustrative design of its temporal sequence, candidate miRNA combinations, and sustained-release strategies (**Table 3**), which broadly divides periodontal repair into three phases: the inflammation-regulation phase (approximately 0–7 days post-surgery), the osteogenic–vascular coupling and regeneration phase (approximately 7–28 days post-surgery), and the tissue maturation and remodeling phase (approximately 1–6 months post-surgery). (These time divisions constitute an engineering working hypothesis based on periodontal wound-healing studies (139); the actual windows should be calibrated according to defect type, infection burden, animal species, and scaffold degradation rate).

**Table 3:** A theoretical spatiotemporally coordinated model for periodontal regeneration.

Dimension	Phase 1: inflammation regulation phase	Phase 2: osteogenic–vascular coupling and regeneration phase	Phase 3: tissue maturation and remodeling phase
Time window	Approx. 0–7 days post-surgery	Approx. 7–28 days post-surgery	Approx. 1–6 months post-surgery
Microenvironmental characteristics	Neutrophils and pro-inflammatory macrophages; elevated levels of TNF-α, IL-1β, IL-6, and ROS	Resolution of inflammation; proliferative repair phase; osteogenic differentiation, ECM deposition, and neovascularization occur simultaneously	Oriented collagen alignment and mineralization under functional loading; reattachment of periodontal ligament fibers and trabecular bone remodeling
Candidate miRNAs	• miR-146a → targets IRAK1, inhibits pro-inflammatory factors • miR-27a-5p → targets PTEN, inhibits bacteria-induced inflammation	• miR-21 → promotes osteogenesis via the PI3K/AKT/GSK3β/β-catenin pathway • miR-210 → upregulates VEGF, potentially involved in the angiogenesis–osteogenesis coupling	• miR-21 → targets ACVR2B to promote osteogenesis under mechanical stimulation; the PI3K/AKT pathway continuously promotes bone formation • miR-26a → the RUNX1/miR-26a/JAG1 axis inhibits osteoclastogenesis
Delivery systems and kinetics	Chitosan/sodium β-glycerophosphate/gelatin thermosensitive hydrogel outer layer + MMP-responsive crosslinker; anti-inflammatory miRNAs encapsulated in cationic polymer nanoparticles; rapid release over several days	PLGA nanocarrier middle layer; controlled molecular weight and LA/GA ratio; sustained release over several weeks	3D-printed β-TCP/HA ceramic core + polymeric sustained-release coating; sustained release over several weeks to several months; mechanical support for transmitting occlusal forces

This model is a prospective theoretical framework derived from integration of existing research evidence; the miRNA combinations are illustrative designs. miR-21 is applied across successive stages, with its functional orientation determined by microenvironment-guided dominant target selection.

Phase 1 (inflammation regulation, approximately 0–7 days post-surgery). This phase is characterized by an acute wound inflammatory response, with extensive infiltration of neutrophils and pro-inflammatory macrophages at the injury site and elevated levels of pro-inflammatory cytokines and reactive oxygen species (ROS). The primary objective is to suppress excessive inflammation and establish a local microenvironment conducive to subsequent regeneration. In this model, the scaffold surface layer consists of a rapidly degradable chitosan/sodium β-glycerophosphate/gelatin thermosensitive hydrogel incorporating a matrix metalloproteinase (MMP)-responsive crosslinker; anti-inflammatory miRNAs encapsulated in cationic polymer nanoparticles are dispersed within the hydrogel. MMPs highly expressed in the inflammatory microenvironment trigger crosslink cleavage, enabling rapid release within days to match the inflammation-regulation phase. Candidate anti-inflammatory miRNAs for this phase include miR-146a, which suppresses pro-inflammatory cytokine secretion by targeting *IRAK1* in an inflammatory environment, and miR-27a-5p, which suppresses bacterially induced inflammatory responses by targeting *PTEN*.

Phase 2 (osteogenic–vascular coupling and regeneration, approximately 7–28 days post-surgery). As inflammation gradually resolves, the wound enters the proliferative repair phase, and local macrophages shift from a predominantly pro-inflammatory state toward reparative and regenerative states (see Section 3.2.1). Beyond secreting anti-inflammatory and reparative factors such as IL-10, they help establish a growth factor-enriched repair microenvironment through intercellular communication with other cells. Osteoblast differentiation, extracellular matrix deposition, and neovascularization proceed simultaneously, making this a critical window for periodontal tissue reconstruction. The middle scaffold layer incorporates poly(lactic-co-glycolic acid) (PLGA) nanocarriers loaded with osteogenic and pro-angiogenic miRNAs; adjustment of polymer molecular weight and the lactide/glycolide (LA/GA) ratio enables sustained release over several weeks to match this phase. Candidate miRNAs include miR-21, which promotes osteogenic differentiation through the PI3K/AKT/GSK3β/β-catenin pathway, and miR-210, which upregulates VEGF expression.

Phase 3 (tissue maturation and remodeling, approximately 1–6 months post-surgery). This phase is characterized by tissue maturation and remodeling, during which regenerated tissue undergoes ordered alignment and mineralization under functional loading (e.g., occlusal forces), leading to periodontal ligament fiber reattachment and trabecular bone remodeling. The inner scaffold layer consists of a three-dimensionally printed β-TCP/hydroxyapatite (HA) ceramic core coated with a polymeric sustained-release film loaded with functional miRNAs; slow degradation of the ceramic matrix provides release over weeks to months. The mechanical support of the ceramic core simultaneously transmits physiological occlusal forces, promoting oriented alignment and mineralization of regenerated tissue through mechanotransduction. Mechanosensitive miRNAs in this phase include miR-21, which responds to mechanical tensile stimulation and regulates osteogenic differentiation of human PDLSCs by targeting *ACVR2B* (activin type IIB receptor), and miR-26a, for which the RUNX1/miR-26a/JAG1 axis inhibits osteoclastogenesis under compressive conditions.

The three-phase candidate miRNA combinations above are theoretical examples that may be replaced or expanded in the future according to specific research contexts and high-throughput screening results. The cross-stage use of miR-21, in particular, illustrates the principle of functional transition of a single molecule across repair phases: its net effect may depend on the microenvironmental context. In Phase 2, growth-factor accumulation in the defect activates the PI3K/AKT pathway, and miR-21 then exerts primarily osteogenic rather than anti-osteogenic effects (see Section 3.1). In Phase 3, superimposed mechanical stimulation directs it to target *ACVR2B*, further enhancing osteogenesis (37). Although this microenvironment-driven “dominant target selection” mechanism requires further experimental validation, it embodies the core value of the model’s temporally controlled release design—namely, aligning with the natural rhythm of tissue repair so that exogenous miRNAs exert their intended dominant regenerative effects at specific phases.

To evaluate the performance of this delivery system, we propose a multi-level evaluation framework encompassing *in vitro* release kinetics, *in vitro* biological functional validation, and *in vivo* tracking and efficacy assessment. At the *in vitro* level, the dialysis bag method or a constant-temperature shaking method would first be used, with key monitoring points at days 1, 3, 7, 14, 28, 56, and 90, to quantify the release behavior of fluorescently labeled miRNAs and characterize the temporal release kinetics corresponding to the three-phase window. Pathway-specific reporter cell lines and functional assays would then be used to verify the biological activity of the released agents in a phase-specific manner. In Phase 1 (inflammation regulation), the pathway-inhibitory efficacy of anti-inflammatory miRNAs would be verified using an LPS-stimulated NF-κB luciferase reporter system. In Phase 2 (osteogenic–vascular coupling and regeneration), osteogenic activity would be assessed with a RUNX2 reporter system combined with ALP activity and alizarin red staining, supplemented by endothelial tubule-formation assays to evaluate pro-angiogenic effects. In Phase 3 (tissue maturation and remodeling), the osteogenic and anti-osteoclastic effects of miRNAs under mechanical loading would be evaluated using a Flexcell loading system together with tartrate-resistant acid phosphatase (TRAP) staining and osteogenic marker assays. At the *in vivo* level, fluorescently labeled miRNA mimics combined with *in vivo* imaging would first be used to monitor overall release trends; tissue sections collected at successive time points would be examined by fluorescence microscopy for residual drug and distribution across scaffold layers to elucidate *in vivo* temporal release patterns. Histological efficacy would then be evaluated in three phases: in Phase 1, local inflammatory cytokine levels and macrophage functional-state distribution would be assessed; in Phase 2, neovascularization and early osteogenesis would be evaluated by histology and immunohistochemistry; and in Phase 3, micro-CT would be used to quantify bone volume fraction (BV/TV), bone mineral density (BMD), and trabecular microstructural parameters, combined with histological assessment of periodontal ligament fiber reattachment and functional cementum–alveolar bone healing, thereby validating the potential of this sequential-release strategy for temporally coordinated synergistic therapy from an integrated perspective.

In summary, this review seeks to overcome the fragmentation of existing research on miRNA-mediated regulation of periodontal immune homeostasis and tissue regeneration and has preliminarily established an integrated analytical framework spanning molecular regulatory networks, smart delivery systems, and temporally coordinated regeneration models. The proposed spatiotemporally coordinated model offers a new theoretical basis for shifting the field from single-target intervention toward multi-network temporal regulation. With the continued refinement of multi-omics technologies, smart biomaterials, and large-animal validation systems, miRNA-based immunomodulatory therapies are expected to accelerate the translation from basic research to clinical application, opening new therapeutic avenues for functional periodontal tissue regeneration.
