# Supplementary material for: MicroRNA-driven periodontal immune homeostasis and tissue regeneration: from regulatory networks to engineering translation
Source: Front Immunol. 2026 Sep 22;17:1960089. doi: 10.3389/fimmu.2026.1960089 (PMC13639849; doi:10.3389/fimmu.2026.1960089)
Supplement: Supplementary file 1 [file SupplementaryFile1.docx]

**Supplementary Material**

This supplementary material provides a detailed description of the literature search and selection strategy and contains five supplementary tables that systematically organize detailed data on the microRNA (miRNA) regulatory networks discussed in this review, including mechanosensitive miRNAs, osteogenic/resorptive differentiation, inflammation-regulated miRNAs, competing endogenous RNA (ceRNA) networks, and miRNAs regulating periodontal tissue homeostasis in extracellular vesicles. The contents are as follows:

**1. Literature Search and Selection Strategy**

This article is a narrative review, and the literature search and selection followed the strategy described below.

**1.1 Databases and search period.**

A systematic search was conducted in the PubMed and Scopus databases, covering the period from January 2015 to July 2026. For seminal works published before 2015 and for highly relevant studies from adjacent fields (e.g., bone tissue engineering, angiogenesis, immunomodulation) whose findings are transferable to periodontal immune homeostasis and regeneration, literature was manually supplemented through two approaches: (i) backward citation searching of the reference lists of included articles; and (ii) targeted manual searches by specific topics or keywords as required by the narrative, to ensure the comprehensiveness and historical continuity of the review.

**1.2 Search terms and Boolean logic.**

The search strategy was built around the core theme of this review, using the following keyword combination:

("microRNA" OR "miRNA" OR "miR") AND ("periodontitis" OR "periodontal tissue" OR "periodontal ligament" OR "periodontal regeneration" OR "alveolar bone" OR "periodontal stem cells" OR "PDLSCs") AND ("osteogenesis" OR "osteogenic differentiation" OR "osteoclast" OR "bone resorption" OR "differentiation" OR "inflammation" OR "immune" OR "immunomodulation" OR "macrophage" OR "ceRNA" OR "angiogenesis" OR "extracellular vesicle" OR "exosome" OR "delivery" OR "scaffold" OR "biomaterial" OR "tissue engineering")

The search string was adjusted according to the syntax rules of each database.

**1.3 Inclusion Criteria**

(i) Studies involving periodontal tissues (periodontal ligament stem cells, alveolar bone, periodontal ligament, cementum, gingiva, etc.), periodontal inflammatory/immune processes, or oral tissue-derived stem cells highly relevant to periodontal regeneration (dental pulp stem cells, stem cells from human exfoliated deciduous teeth, dental follicle stem cells, etc.); or studies addressing bone metabolism, inflammation and immunomodulation, angiogenesis, and extracellular vesicles whose molecular mechanisms or engineering strategies have clear reference value for periodontal regeneration.

(ii) Studies involving miRNA expression regulation, biological functions, molecular mechanisms, or engineered delivery. Background literature essential for the narrative (e.g., overviews of periodontal disease, fundamentals of bone biology and immunology, nucleic acid delivery and biomaterials, omics technologies and methods) was included as appropriate. Cross-disciplinary literature with transferable conceptual analogies or parallel reference value for periodontal regeneration mechanisms or strategies (e.g., multi-target regulation by natural products, immunomodulation in systemic diseases) could be included as background literature or conceptual introduction, but was not part of the core mechanistic discussion.

(iii) Study types included original research (in vitro cell experiments, in vivo animal experiments, clinical studies) and reviews.

(iv) Published in peer-reviewed journals.

**1.4 Exclusion Criteria**

(i) Preliminary studies involving only miRNA expression profiling without functional or mechanistic validation were not included in the core mechanistic discussion and could only be cited as background evidence of expression.

(ii) Pure tumor or other organ studies with no direct relevance to periodontal tissues and no transferability.

(iii) Conference abstracts, theses, editorials, and non-peer-reviewed preprints.

(iv) For duplicate publications, only the most recent or most complete version was included.

**1.5 Literature Screening Process**

After the initial search, titles and abstracts were first screened to exclude clearly irrelevant articles; full texts were then retrieved for secondary screening, and final inclusion was determined according to the inclusion/exclusion criteria. For studies with discrepancies, two investigators independently evaluated them and reached a consensus through discussion. More than 200 articles were ultimately included in this review, covering multiple dimensions such as miRNA regulation of periodontal immune homeostasis, osteogenic differentiation, angiogenesis, extracellular vesicle communication, and engineered delivery. In addition, literature not covered by the systematic search but highly relevant to the narrative (including general mechanism studies of immunity/angiogenesis from non-periodontal sources, methodological and technical literature, background reviews on the association between oral health and systemic diseases, etc.) was supplemented through targeted manual searches and backward citation tracing.

**2. Supplementary Tables**

**Supplementary Table S1. Mechanisms of action of mechanosensitive miRNAs in periodontal tissue remodeling**

**Supplementary Table S2. miRNAs regulating osteogenic/resorptive differentiation in periodontal tissues and their molecular mechanisms**

**Supplementary Table S3. miRNAs mediating the regulation of periodontal inflammation and their molecular mechanisms**

**Supplementary Table S4. Regulatory network of circRNA/lncRNA–miRNA–signal pathways in periodontal tissues**

**Supplementary Table S5. miRNAs regulating periodontal tissue homeostasis in extracellular vesicles and their molecular mechanisms**

**Supplementary Table S1. Mechanisms of action of mechanosensitive miRNAs in periodontal tissue remodeling**

This table summarizes 17 groups of miRNAs regulated by mechanical forces (fluid shear stress, tensile stress, compressive stress). It systematically organizes the corresponding cell models, types of mechanical responses, expression alterations, molecular mechanisms, and functional effects of these miRNAs, focusing on their regulatory roles in osteogenic/osteoclastic differentiation, inflammatory and immune responses, as well as cell proliferation and apoptosis.

| **No.** | **miRNA** | **Cell** **model** | **Mechanical** **response** | **Expression** **change** | **Molecular** **mechanism** | **Functional** **effect** | **References** |
| --- | --- | --- | --- | --- | --- | --- | --- |
| 1 | miR-20a | MC3T3-E1 | Fluid shear stress | Upregulated | Targets *BAMBI* and *SMAD6,* activating the BMP/SMAD pathway | Promotes osteogenesis | (1) |
| 2 | miR-21 | PDLSCs/MC3T3-E1 | Tensile force | Upregulated | Targets *PI3K/AKT/GSK3β* pathway, *SMAD7* | Promotes osteogenesis and mineralization | (2–5) |
| 3 | miR-34a | PDLSCs/MC3T3-E1 | Tensile force/fluid shear stress | Downregulated | Targets *CELF3,* *DKK1,* *FGFR1,* *GSK-3β* | Downregulation under mechanical stimulation promotes osteogenesis; upregulation by drug induction also promotes osteogenesis | (6–9) |
| 4 | miR-132 | PDLCs | Fluid shear stress | Upregulated | Activates the PI3K/AKT/mTOR pathway | Promotes proliferation and differentiation | (10) |
| 5 | miR-33-5p | MC3T3-E1 | Tensile force/fluid shear stress | Altered | Targets *Hmga2* | Promotes bone formation | (11,12) |
| 6 | miR-1246 | PDLSCs/TMJ condylar chondrocytes | Cyclic tensile force; M1 macrophage-derived exosomes | Upregulated | Inhibits *GSK3β* and *Axin2,* activates the Wnt/β-catenin pathway | Promotes osteogenic differentiation; promotes inflammation | (13,14) |
| 7 | miR-195-5p | PDLCs | Cyclic tensile force | Downregulated | Targets *WNT3A,* *FGF2,* *BMPR1A;* downregulation relieves inhibition | Downregulation promotes osteogenesis | (15,16) |
| 8 | miR-103a | hFOB1.19 | Cyclic tensile force | Downregulated | Targets *RUNX2* | Downregulation promotes osteogenesis | (17) |
| 9 | miR-138-5p | Osteoblasts | Cyclic tensile force | Downregulated | Targets *MACF1;* downregulation activates Wnt/BMP signaling | Downregulation promotes osteogenesis | (18) |
| 10 | miR-26a | PDLCs | Compressive force | Downregulated | RUNX1/miR-26a/JAG1 axis | Inhibits osteoclastogenesis | (19) |
| 11 | miR-132-3p | MC3T3-E1 | Cyclic tensile force | Upregulated | Targets *SMAD5,* inhibits BMP signaling | Inhibits osteogenic differentiation | (20) |
| 12 | miR-503-5p | BMSCs | Cyclic tensile force | Downregulated | Targets *SORBS1,* *RUNX2* | Inhibits osteogenesis | (21,22) |
| 13 | miR-3198 | PDLCs | Tensile force/compressive force | Tension: downregulated; compression: upregulated | Targets *OPG,* reduces the OPG/RANKL ratio | Promotes bone resorption | (23) |
| 14 | miR-146a-5p | Osteoclasts/PDLSCs | Compressive force; tensile force | Downregulated | Targets *adiponectin;* targets *CELF3* | Stimulates angiogenesis; inhibits osteogenesis | (6,24) |
| 15 | miR-140-5p | MC3T3-E1 | Fluid shear stress | Downregulated | VEGFA/ERK5 signaling pathway | Downregulation promotes osteoblast proliferation | (25) |
| 16 | miR-214-3p | Osteoblasts | Fluid shear stress | Downregulated | Targets *ATF4* | Downregulation promotes proliferation | (26) |
| 17 | miR-494-3p | MC3T3-E1 | Compressive force | Upregulated | Targets *FGFR2,* *ROCK1* | Inhibits proliferation | (27) |

**Supplementary Table S2. miRNAs regulating osteogenic/osteoclastic differentiation in periodontal tissues and their molecular mechanisms**

This table systematically summarizes 38 groups of miRNAs regulating osteogenic/osteoclastic differentiation in periodontal tissues, covering their source cell types, target molecules, molecular mechanisms, and functional effects. It provides comprehensive molecular evidence for understanding the central role of miRNAs in the balance of periodontal bone metabolism.

| **No.** | **miRNA** | **Source** **cell** **type** | **Molecular** **mechanism** | **Functional** **effect** | **References** |
| --- | --- | --- | --- | --- | --- |
| 1 | miR-100 | Adipose-derived MSCs | Targets *BMPR2* | Downregulation promotes osteogenic differentiation; overexpression inhibits osteogenic differentiation | (28) |
| 2 | miR-125b | PDLSCs | Targets *NKIRAS2* and enhances NF-κB pathway activity | Inhibits osteogenic differentiation of PDLSCs | (29) |
| 3 | miR-132 | PDLSCs | Targets *GDF5* and activates the NF-κB axis | Inhibits osteogenic differentiation of PDLSCs | (30) |
| 4 | miR-138 | PDLSCs | Targets OCN | Inhibits mineralization | (31) |
| 5 | miR-141-3p | Stem cells from the apical papilla (SCAP) | Downregulates *SPAG9* and activates the MAPK pathway | Promotes osteogenic differentiation | (32) |
| 6 | miR-142-3p | PDLSCs | Targets *SGK1* | Inhibits osteogenic differentiation of PDLSCs | (33) |
| 7 | miR-146a | PDLSCs; gingival fibroblasts; BMSCs | Inhibits the NF-κB pathway; targets *IRAK1;* targets PGE2 synthase | Promotes osteogenesis and inhibits osteoclastogenesis; inhibits inflammatory responses and regulates immune homeostasis | (34–41) |
| 8 | miR-15b | BMSCs | Targets *SMURF1,* protecting *RUNX2* protein from degradation | Promotes osteogenic differentiation | (42) |
| 9 | miR-152-3p | hPDLSCs | Targets *ITGA5* | Downregulation promotes hPDLSC proliferation and osteogenic differentiation | (43) |
| 10 | miR-17/miR-17-5p | PDLSCs; MSCs | Downregulated expression under inflammatory conditions leads to *SMURF1* activation; targets *SMAD7* | Promotes osteogenesis at normal levels; low expression under inflammatory conditions impairs osteogenesis | (44,45) |
| 11 | miR-155 | Bone marrow-derived monocytes | Targets *SOCS1* | Inhibits osteoclast differentiation | (46) |
| 12 | miR-184 | PDLSCs | Targets *NFI-C* | Inhibits osteogenic differentiation of PDLSCs | (47) |
| 13 | miR-194 | BMSCs | Targets *COUP-TFII* | Promotes osteogenic differentiation | (48) |
| 14 | miR-195-5p | BMSCs | Targets *WNT3A,* downregulating *RUNX2* and osteopontin | Inhibits osteogenesis | (49) |
| 15 | miR-199a-5p | Stem cells from the apical papilla (SCAP) | Targets *IFIT2* | Enhances cell proliferation and osteogenic differentiation | (50) |
| 16 | miR-200a-3p | PDLSCs; BMSCs | Targets *ZEB2* and activates NF-κB; FOXJ1/NF-κB/MMP axis | Inhibits osteogenesis in PDLSCs; promotes BMSC migration | (51,52) |
| 17 | miR-200c | BMSCs | Targets *SOX2* and *KLF4,* relieving the inhibitory effect of *SOX2* on Wnt signaling | Promotes osteogenic differentiation of BMSCs | (53) |
| 18 | miR-21 | MC3T3-E1; PDLSCs; hUMSCs; BMSCs; T cells | Facilitates expression of key osteogenic factors (ALP, *RUNX2,* osteopontin, osterix, *MEF2C);* targets *ACVR2B* under mechanical stretch; activates PI3K/AKT/GSK3β pathway and stabilizes β-catenin; targets *SMAD7;* targets *SMAD5;* acts through PTEN/AKT/TGF-β1 pathway; modulates RANKL/OPG balance | Promotes osteogenesis; inhibits osteogenesis; enhances inflammatory response; promotes orthodontic tooth movement | (2,3,5,54–60) |
| 19 | miR-210 | PDLSCs | Regulates VEGF expression and promotes angiogenesis | Promotes angiogenesis and associated osteogenesis | (61) |
| 20 | miR-214 | PDLSCs | Regulates the Wnt/β-catenin signaling pathway | Inhibits osteogenesis | (62) |
| 21 | miR-22 | PDLSCs | Targets *HDAC6* | Promotes osteogenic differentiation of PDLSCs | (63) |
| 22 | miR-223 | Periodontal ligament-derived cells | Targets *FGFR2* and *TGFβR2* | Inhibits osteogenesis | (64) |
| 23 | miR-23a | PDLSCs | Targets *BMPR1B* to inhibit *SMAD1/5/9* phosphorylation | Inhibits osteogenic differentiation of PDLSCs | (65) |
| 24 | miR-23b | PDLSCs | Targets *RUNX2* | Inhibits osteogenesis | (66) |
| 25 | miR-24-3p | PDLSCs | Targets *SMAD5* | Downregulation promotes osteogenesis | (67) |
| 26 | miR-27 | MDPC-23 | Targets *APC* and *SFRP1,* activating the Wnt pathway | Promotes odontoblastic differentiation; promotes periodontal regeneration via osteogenesis/angiogenesis | (68,69) |
| 27 | miR-29 family | Osteoblasts; osteoclasts | Targets *DKK1,* *KREMEN2,* and *SFRP2,* activating the Wnt pathway; targets *CALCR* | Promotes osteogenic differentiation; may be involved in osteoclastic regulation | (70,71) |
| 28 | miR-31 | BMSCs | Targets osterix *(SP7);* reduces expression of OPN, BSP, OSX, and OCN, but not *RUNX2;* represses *SATB2* protein levels | Inhibits osteogenesis | (72,73) |
| 29 | miR-34a | PDLSCs; MSCs | Targets *CELF3* and JAG1/Notch; targets DKK1/Wnt | Bidirectional: inhibits osteogenesis; promotes osteogenesis | (6–8,74) |
| 30 | miR-375 | PDLSCs | Targets *TOB2* | Promotes proliferation and osteogenic differentiation of PDLSCs | (75) |
| 31 | miR-383-5p | PDLSCs | Targets *HDAC9* | Promotes osteogenic differentiation of PDLSCs | (76) |
| 32 | miR-589-3p | PDLSCs | Targets *ATF1* | Promotes osteogenesis | (77) |
| 33 | miR-628-5p | MSCs | Upregulated following silencing of *ETV1,* *SOX11,* and *GATA6* | Inhibits osteogenesis | (78) |
| 34 | miR-338 cluster | Osteoclasts | Regulates the *STAT1* pathway, influencing M1 polarization and osteoclast differentiation | Ablation of the Mir338 cluster inhibits osteoclastogenesis | (79) |
| 35 | miR-508-5p | hPDLSCs | Targets *SOX11* | Inhibits osteogenesis | (80) |
| 36 | miR-320c; miR-372/373-3p | hPDLSCs | Downregulation relieves suppression of *RUNX2* | Downregulation promotes osteogenesis | (81) |
| 37 | miR-584-5p | PDLSCs | Upregulates *H2AFZ* and suppresses *RUNX2* | Inhibits osteogenic differentiation | (82) |
| 38 | let-7a | MSCs | Targets FAS/FASL | Promotes osteogenesis | (83) |

**Supplementary Table S3. miRNAs mediating the regulation of periodontal inflammation and their molecular mechanisms**

This table summarizes 15 groups of miRNAs that regulate the periodontal inflammatory response, covering their cellular origins, expression profiles, target molecules and signaling pathways, and functional effects. It provides a reference for the molecular mechanisms underlying the regulatory roles of miRNAs in the initiation, progression, and resolution of periodontal inflammation.

| **No.** | **miRNA** | **Source** **cell** **type** | **Expression** **change** | **Molecular** **mechanism** | **Functional** **effect** | **References** |
| --- | --- | --- | --- | --- | --- | --- |
| 1 | miR-105 | Gingival keratinocytes | Upregulated upon bacterial stimulation | Targets *TLR2* | Attenuates the inflammatory response | (84) |
| 2 | miR-128 | THP-1-derived macrophages; periodontitis tissue | Upregulated in periodontitis/inflammation | Acts via the p38 signaling pathway and attenuates the TNF-α response | Modulates the inflammatory response | (85) |
| 3 | miR-128-3p | BMSCs | Upregulated upon TNF-α induction | Targets *SIRT1* | Exacerbates the inflammatory response | (86) |
| 4 | miR-181a-5p | BMSCs | Upregulated in experimental periodontitis | IL-6/STAT3 signaling axis mediates miR-181a-5p upregulation | Promotes Th17/Treg immune imbalance and aggravates alveolar bone resorption | (87) |
| 5 | miR-212-5p | PDLSCs | Significantly downregulated upon LPS treatment | Targets *MyD88* | Overexpression attenuates LPS-induced inflammatory responses | (88) |
| 6 | miR-20a; miR-30e; miR-93 | Gingival mesenchymal stem cells (GMSCs) | Upregulated in periodontitis | Regulate gene networks involved in inflammatory and hypoxic responses | Involved in the regulation of immune responses and hypoxia adaptation | (89) |
| 7 | miR-203 | Gingival epithelial cells | Upregulated upon stimulation by P. gingivalis | Inhibits *SOCS3* and activates *STAT3* | Regulates epithelial cell immune responses | (90) |
| 8 | miR-28-5p | PDLSCs; gingival crevicular fluid | Downregulated in LPS-stimulated PDLCs | Targets *SPHK1* | Downregulation leads to elevation of *SPHK1,* exacerbating inflammatory responses | (91) |
| 9 | miR-495-3p; miR-539-5p; miR-34c-3p; miR-7a-2-3p | Periodontal ligament cells | Significantly downregulated upon LPS stimulation | The mechanism requires further clarification | Involved in the regulation of stress responses | (92) |
| 10 | miR-21-5p; miR-498; miR-548a-5p | Periodontal ligament cells | Significantly upregulated upon LPS stimulation | The mechanism requires further elucidation | Involved in the regulation of stress responses | (92) |
| 11 | miR-221; miR-222-3p | PDLCs; rat periodontitis model | Downregulated in LPS-stimulated PDLCs and in periodontitis rat tissues | LPS activates the STAT1 pathway and suppresses miR-221/222-3p expression; miR-221/222-3p targets *PAK1* | Downregulation leads to exacerbated inflammation and bone destruction | (93) |
| 12 | miR-27a-5p | Animal models of periodontitis | Downregulated under inflammatory/bacterial stimulation | Targets *PTEN* | Alleviates the inflammatory response | (94) |
| 13 | miR-543-3p | PDLSCs | Downregulated upon LPS treatment | Targets *KLF6* | Downregulates inflammation | (95) |
| 14 | miR-584 | Gingival epithelial cells | Upregulated upon P. gingivalis stimulation | Inhibits the lactoferrin receptor | Exacerbating the inflammatory response | (96) |
| 15 | miR-671-5p | PDLSCs | Significantly upregulated upon LPS treatment | Targets *DUSP8* in the p38 MAPK pathway | Inhibits proliferation and enhances pro-inflammatory responses | (97) |

**Supplementary Table S4. Regulatory network of circRNA/lncRNA–miRNA–signaling pathways in periodontal tissues**

This table summarizes 24 circRNA/lncRNA–miRNA–mRNA regulatory axes, illustrating the ceRNA mechanism whereby circRNAs and lncRNAs act as molecular sponges to bind miRNAs, thereby relieving miRNA-mediated repression of downstream target genes. It elucidates the molecular mechanisms by which these ceRNAs regulate osteogenic differentiation of periodontal stem cells and the inflammatory microenvironment.

| **No.** | **ceRNA** **type** | **ceRNA** **name** | **Target** **miRNA** | **Molecular** **mechanism** | **Functional** **effect** | **References** |
| --- | --- | --- | --- | --- | --- | --- |
| 1 | circRNA | circSKIL | miR-532-5p | Acts as a ceRNA to bind miR-532-5p, relieving its repression of *NOTCH1* and activating the Notch signaling pathway | Promotes osteogenic differentiation of hPDLCs | (98) |
| 2 | circRNA | circFAT1 | miR-4781-3p | Binds miR-4781-3p, relieving its repression of *SMAD5* and activating the BMP/SMAD pathway | Promotes osteogenic differentiation and mineralization of PDLSCs | (99) |
| 3 | circRNA | circCDR1as | miR-7 | Binds miR-7, relieving its repression of *GDF5* and activating *SMAD1/5/8* and the p38 MAPK pathway | Promotes osteogenic differentiation of PDLSCs | (100) |
| 4 | circRNA | circ-SPATA13 | miR-485-5p_R+1 | Binds miR-485-5p_R+1, relieving its repression of BMP | Promotes osteogenic differentiation of PDLSCs | (101) |
| 5 | circRNA | circBIRC6 | miR-543 | Binds miR-543, relieving its repression of *PTEN;* knockdown of circBIRC6 increases miR-543 levels, decreases *PTEN,* and activates PI3K/AKT/mTOR signaling to promote osteogenesis | Absence of circBIRC6 promotes osteogenesis in PDLSCs; high expression may inhibit osteogenesis | (102) |
| 6 | circRNA | circ_0054633 | miR-590-3p | Forms a circ_0054633/miR-590-3p/*RUNX2* positive feedback loop: *RUNX2* promotes circ_0054633 transcription, and circ_0054633 sequesters miR-590-3p to relieve its repression of *RUNX2* | Promotes osteogenic differentiation of BMSCs | (103) |
| 7 | circRNA | mmu_circ_0000217 | miR-3064-3p | Binds miR-3064-3p, relieving its repression of *Dkk1* and activating the JAK/STAT3 pathway | Promotes osteogenic differentiation of OCCM-30 cells | (104) |
| 8 | circRNA | circ_0003072 | miR-488-3p | Binds miR-488-3p, relieving its repression of *CHRDL1* | Promotes osteogenic differentiation of PDLSCs | (105) |
| 9 | circRNA | circ_0036872 | miR-143-3p | Regulates the miR-143-3p/*IGF2* axis | Enhances osteogenesis of dental pulp stem cells | (106) |
| 10 | circRNA | circ_0007349 | miR-127-5p | Sponges miR-127-5p to regulate glutamate-ammonia ligase *(GLUL)* | Promotes gingival fibroblast proliferation, migration, wound healing, and extracellular matrix turnover | (107) |
| 11 | lncRNA | FER1L4 | miR-874-3p | Binds miR-874-3p, relieving its repression of *VEGFA* | Promotes osteogenic differentiation of PDLSCs and in vivo bone formation | (108) |
| 12 | lncRNA | JHDM1D-AS1 | miR-532-5p | Binds miR-532-5p, relieving its repression of *IGF1R* and activating the Notch/*HES1* pathway | Promotes osteogenic differentiation of hPDLCs | (109) |
| 13 | lncRNA | PWAR6 | miR-106a-5p | Binds miR-106a-5p, relieving its repression of *BMP2* | Promotes osteogenic differentiation and mineralization of hPDLSCs | (110) |
| 14 | lncRNA | MEG3 | miR-27a-3p | Binds miR-27a-3p, relieving its repression of *IGF1* | Overexpression of MEG3 promotes osteogenesis | (111) |
| 15 | lncRNA | MEG8 | miR-495-3p; miR-485-3p | Binds miR-495-3p and miR-485-3p via the ceRNA mechanism, modulating *TCF4* expression and activating the Wnt/β-catenin pathway | Promotes osteogenic differentiation of PDLSCs | (112) |
| 16 | lncRNA | SNHG5 | miR-23b-3p | Binds miR-23b-3p, relieving its repression of *RUNX2* | Promotes osteogenic differentiation of PDLSCs | (113) |
| 17 | lncRNA | PVT1 | miR-10a-5p | Binds miR-10a-5p, relieving its repression of *BDNF* | Promotes osteogenic differentiation of PDLCs | (114) |
| 18 | lncRNA | MALAT1 | miR-155-5p | Binds miR-155-5p, relieving its repression of *ETS1* | Promotes osteogenic differentiation of PDLSCs | (115) |
| 19 | lncRNA | MALAT1 | miR-93-5p | Binds miR-93-5p, relieving its repression of *SMAD5* | Promotes osteogenic differentiation of PDLSCs | (116) |
| 20 | lncRNA | MALAT1 | miR-124-3p | Binds miR-124-3p, relieving its repression of *IGF2BP1* | Promotes osteogenic differentiation of PDLSCs | (117) |
| 21 | lncRNA | MALAT1 | miR-30b | Binds miR-30b, regulating macrophage polarization | Promotes inflammation | (118) |
| 22 | lncRNA | LINC00707 | miR-490-3p | Binds miR-490-3p, thereby upregulating *FOXO1* | Promotes osteogenic differentiation of PDLSCs | (119) |
| 23 | lncRNA | LINC00968 | miR-3658 | Binds miR-3658, relieving its repression of *RUNX2* | Promotes osteoblast differentiation of dental pulp stem cells and bone formation | (120,121) |
| 24 | lncRNA | SNHG1 | miR-9-5p | Binds miR-9-5p, relieving its repression of NF-κB | Enhances the inflammatory response | (122) |

**Supplementary Table S5. miRNAs mediating periodontal tissue homeostasis via extracellular vesicles and their molecular mechanisms**

This table systematically compiles 34 groups of miRNAs carried by extracellular vesicles that participate in regulating periodontal tissue homeostasis. They are categorized into seven major functional groups: osteogenesis promotion, osteogenesis inhibition, inflammatory and immune regulation, angiogenesis regulation, resorption regulation, dental-pulp repair, and physiological processes. The table details their cellular sources/vesicle types, molecular mechanisms, and functional effects.

| **Category** | **No.** | **miRNA** | **Source** **cell/vesicle** **type** | **Molecular** **mechanism** | **Functional** **effect** | **References** |
| --- | --- | --- | --- | --- | --- | --- |
| Osteogenesis promotion | 1 | miR-22-3p | BMSCs (EVs) | Inhibits *FTO,* inactivating the MYC/PI3K/AKT pathway | Promotes osteogenic differentiation of BMSCs | (123) |
|  | 2 | miR-181d-5p | Periodontal ligament cells (exosomes; cyclic stretching-induced) | Inhibits *TNF* | Promotes osteoblast differentiation | (124) |
|  | 3 | miR-26a | BMSCs (exosomes; DP7-C-transfected) | Associated with the mTOR pathway | Promotes osteogenesis and inhibits bone loss | (125) |
|  | 4 | miR-3679-5p; miR-6747-5p | P2X7R-engineered PDLSCs (exosomes) | Downregulates *GREM1,* activating the BMP pathway | Reverses inflammatory damage and restores the regenerative potential of PDLSCs | (126) |
|  | 5 | miR-122-5p; miR-142-5p; miR-25-3p; miR-192-5p | PDLSCs (exosomes) | Specifically enriched during osteogenic differentiation, synergistically regulating downstream osteogenic target genes | Promotes bone regeneration | (127) |
|  | 6 | miR-758-5p | Inflammatory DPSCs (EVs) | Directly inhibits *LMBR1* and activates the BMP signaling pathway | Enhances osteogenic and odontogenic differentiation of PDLSCs | (128) |
|  | 7 | miR-21b | Macrophages (exosomes) | Targets *SOCS1,* activating the JAK2/STAT3 signaling pathway | Promotes bone regeneration | (129) |
|  | 8 | miR-494-3p | Macrophages (exosomes) | Targets *CAMK2D* | Promotes osteogenic differentiation of hPDLSCs | (130) |
|  | 9 | miR-451a | DPSCs (small EVs) | Activates AKT/eNOS signaling pathways | Induces vascularized bone regeneration | (131) |
|  | 10 | miR-5107-5p | Macrophages (EVs) | Suppresses *EGFR* expression and alleviates its inhibitory effect on *RhoA* | Restores the osteogenic capacity of mBMSCs under inflammation | (132) |
| Osteogenesis inhibition | 11 | miR-184 | Dental follicle stem cells (LPS-pretreated; exosomes) | Targets *PPARα;* inhibition of miR-184 activates AKT and inhibits JNK | Inhibits osteogenesis; inhibition of miR-184 promotes osteogenic gene expression in PDLSCs | (133) |
|  | 12 | miR-23a | Gingival fibroblasts (exosomes; secreted after radiotherapy) | Targets *CXCL12* | Inhibits osteogenic differentiation of BMSCs | (134) |
|  | 13 | miR-34c-5p | Periodontal ligament fibroblasts (exosomes; PGE2-induced) | Targets *SATB2,* inhibiting ERK pathway phosphorylation | Inhibits osteogenic differentiation of PDLSCs | (135) |
|  | 14 | miR-424-5p | BMSCs (exosomes) | Targets *WIF1,* inhibiting the Wnt/β-catenin signaling pathway | Inhibits osteogenesis | (136) |
|  | 15 | miR-483-5p | Macrophages (apoptotic bodies) | Targets *COL1A1* | Inhibits osteoblast differentiation | (137) |
|  | 16 | let-7c-3p | Periodontal ligament MSCs | Targets NT5E/CD73 | Promotes proliferation and migration of PDLSCs and inhibits their osteogenic differentiation | (138) |
| Resorption regulation | 17 | miR-1260b | MSCs (TNF-α-pretreated; exosomes) | Targets the Wnt5a-mediated RANKL pathway; inhibits *ATF6β,* reducing RANKL expression | Inhibits bone loss | (139,140) |
|  | 18 | miR-31-5p | PDLSCs (exosomes) | Targets eNOS | Inhibits osteoclastogenesis | (141) |
|  | 19 | miR-223-3p | MSCs (apoptotic bodies) | Targets *ITGB1* | Inhibits osteoclastogenesis | (142) |
| Inflammatory and immune regulation | 20 | miR-1246 | MSCs (exosomes/EVs); DPSCs (exosomes) | Targets and inhibits NFAT5/ACE2 | Restores the Th17/Treg balance and alleviates the inflammatory response | (143–145) |
|  | 21 | miR-143-3p | Inflammatory PDLSCs (exosomes); macrophages (apoptotic vesicles) | Targets and inhibits PI3Kγ, regulating the PI3K/AKT/NF-κB axis; targets *IGFBP5* | Promotes M1 macrophage polarization, exacerbating inflammation; induces periodontal bone destruction | (146,147) |
|  | 22 | miR-155-5p | PDLSCs (LPS-pretreated; exosomes) | Targets *SIRT1* | Downregulation restores the Th17/Treg balance and alleviates inflammation | (148) |
|  | 23 | miR-205-5p | PDLSCs (exosomes) | Targets *XBP1* | Downregulation disrupts the Th17/Treg balance and exacerbates inflammation | (149) |
|  | 24 | miR-433-3p | PDLSCs (LPS-pretreated; EVs) | Targets the TLR2/TLR4/NF-κB p65 pathway | Promotes M1 polarization and exacerbates inflammation | (150) |
|  | 25 | miR-100-5p | SHED (exosomes) | Targets and inhibits mTOR, suppressing translation of downstream pro-inflammatory factors | Inhibits inflammation | (151) |
|  | 26 | miR-9-5p | PDLCs (exosomes) | Triggers M1 polarization via the SIRT1/NF-κB signaling pathway in cultured macrophages | Enhances periodontal inflammation | (152) |
|  | 27 | miR-92a-3p | SHED (exosomes) | Inactivates the KLF4/PI3K/AKT signaling pathway | Inhibits periodontitis progression and alleviates pathological damage | (153) |
| Angiogenesis regulation | 28 | miR-378a | Periodontitis-compromised DPSCs (EVs) | Downregulates *SUFU,* activating the Hedgehog/GLI1 pathway | Promotes angiogenesis | (154) |
|  | 29 | miR-125a | Adipose-derived stem cells (ADSCs; exosomes) | Targets and inhibits *DLL4* | Promotes endothelial angiogenesis | (155) |
|  | 30 | miR-423-5p | ADSCs (exosomes) | Targets *SUFU* | Promotes angiogenesis | (156) |
|  | 31 | miR-17-5p | PDLSCs (exosomes) | Targets *VEGFA* | Downregulation promotes angiogenesis | (157) |
| Dental-pulp repair | 32 | miR-27a-5p | DPSCs (exosomes) | Downregulates *LTBP1,* activating the TGFβ1/SMAD signaling pathway | Promotes odontogenic differentiation | (158) |
| Physiological processes | 33 | miR-24-2; miR-142; miR-335; miR-490; miR-296 | PDLSCs (EVs) | Target Ras protein signal transduction; regulate actin/microtubule cytoskeleton organization | May be implicated in silencing of proto-oncogenes | (159) |
|  | 34 | let-7f-5p | Genetically engineered M2-like macrophage-derived exosomes | Downregulates *CKIP-1,* activating PGC-1α-dependent mitochondrial biogenesis | Contributes to cementum regeneration | (160) |

**References**

1.	Peng Z, Mai Z, Xiao F, Liu G, Wang Y, Xie S, et al. MiR-20a: a mechanosensitive microRNA that regulates fluid shear stress-mediated osteogenic differentiation via the BMP2 signaling pathway by targeting BAMBI and SMAD6. Ann Transl Med. 2022 Jun;10(12):683. doi:10.21037/atm-22-2753 PubMed PMID: 35845505; PubMed Central PMCID: PMC9279817.

2.	Wei F, Liu D, Feng C, Zhang F, Yang S, Hu Y, et al. microRNA-21 mediates stretch-induced osteogenic differentiation in human periodontal ligament stem cells. Stem Cells Dev. 2015 Feb 1;24(3):312–9. doi:10.1089/scd.2014.0191 PubMed PMID: 25203845; PubMed Central PMCID: PMC4303015.

3.	Meng YB, Li X, Li ZY, Zhao J, Yuan XB, Ren Y, et al. microRNA-21 promotes osteogenic differentiation of mesenchymal stem cells by the PI3K/β-catenin pathway. J Orthop Res. 2015 Jul;33(7):957–64. doi:10.1002/jor.22884 PubMed PMID: 25728838.

4.	Zhang X, Chen D, Zheng J, Deng L, Chen Z, Ling J, et al. Effect of microRNA-21 on hypoxia-inducible factor-1α in orthodontic tooth movement and human periodontal ligament cells under hypoxia. Exp Ther Med. 2019 Apr;17(4):2830–6. doi:10.3892/etm.2019.7248 PubMed PMID: 30930976; PubMed Central PMCID: PMC6425288.

5.	Li H, Yang F, Wang Z, Fu Q, Liang A. MicroRNA-21 promotes osteogenic differentiation by targeting small mothers against decapentaplegic 7. Mol Med Rep. 2015 Jul;12(1):1561–7. doi:10.3892/mmr.2015.3497 PubMed PMID: 25815684.

6.	Meng X, Wang W, Wang X. MicroRNA-34a and microRNA-146a target CELF3 and suppress the osteogenic differentiation of periodontal ligament stem cells under cyclic mechanical stretch. J Dent Sci. 2022 Jul;17(3):1281–91. doi:10.1016/j.jds.2021.11.011 PubMed PMID: 35784124; PubMed Central PMCID: PMC9236897.

7.	Zeng HB, Dong LQ, Xu C, Zhao XH, Wu LG. Artesunate promotes osteoblast differentiation through miR-34a/DKK1 axis. Acta Histochem. 2020 Oct;122(7):151601. doi:10.1016/j.acthis.2020.151601 PubMed PMID: 33066846.

8.	Wang X, He J, Wang H, Zhao D, Geng B, Wang S, et al. Fluid shear stress regulates osteoblast proliferation and apoptosis via the lncRNA TUG1/miR-34a/FGFR1 axis. J Cell Mol Med. 2021 Sep;25(18):8734–47. doi:10.1111/jcmm.16829 PubMed PMID: 34350720; PubMed Central PMCID: PMC8435422.

9.	Yu W, Zheng Y, Yang Z, Fei H, Wang Y, Hou X, et al. N-AC-l-Leu-PEI-mediated miR-34a delivery improves osteogenic differentiation under orthodontic force. Oncotarget. 2017 Dec 15;8(66):110460–73. doi:10.18632/oncotarget.22790 PubMed PMID: 29299161; PubMed Central PMCID: PMC5746396.

10.	Qi L, Zhang Y. The microRNA 132 regulates fluid shear stress-induced differentiation in periodontal ligament cells through mTOR signaling pathway. Cell Physiol Biochem. 2014;33(2):433–45. doi:10.1159/000358624 PubMed PMID: 24556727.

11.	Guo Y, Wang Y, Liu Y, Liu Y, Zeng Q, Zhao Y, et al. MicroRNA-218, microRNA-191*, microRNA-3070a and microRNA-33 are responsive to mechanical strain exerted on osteoblastic cells. Mol Med Rep. 2015 Aug;12(2):3033–8. doi:10.3892/mmr.2015.3705 PubMed PMID: 25937096.

12.	Wang H, Sun Z, Wang Y, Hu Z, Zhou H, Zhang L, et al. miR-33-5p, a novel mechano-sensitive microRNA promotes osteoblast differentiation by targeting Hmga2. Sci Rep. 2016 Mar 16;6:23170. doi:10.1038/srep23170 PubMed PMID: 26980276; PubMed Central PMCID: PMC4793269.

13.	Wei FL, Wang JH, Ding G, Yang SY, Li Y, Hu YJ, et al. Mechanical force-induced specific MicroRNA expression in human periodontal ligament stem cells. Cells Tissues Organs. 2014;199(5–6):353–63. doi:10.1159/000369613 PubMed PMID: 25823370.

14.	Peng S, Yan Y, Li R, Dai H, Xu J. Extracellular vesicles from M1-polarized macrophages promote inflammation in the temporomandibular joint via miR-1246 activation of the Wnt/β-catenin pathway. Ann N Y Acad Sci. 2021 Nov;1503(1):48–59. doi:10.1111/nyas.14590 PubMed PMID: 33759195.

15.	Chang M, Lin H, Fu H, Wang B, Han G, Fan M. MicroRNA-195-5p Regulates Osteogenic Differentiation of Periodontal Ligament Cells Under Mechanical Loading. J Cell Physiol. 2017 Dec;232(12):3762–74. doi:10.1002/jcp.25856 PubMed PMID: 28181691.

16.	Chang M, Lin H, Luo M, Wang J, Han G. Integrated miRNA and mRNA expression profiling of tension force-induced bone formation in periodontal ligament cells. In Vitro Cell Dev Biol Anim. 2015 Sep;51(8):797–807. doi:10.1007/s11626-015-9892-0 PubMed PMID: 26091625.

17.	Zuo B, Zhu J, Li J, Wang C, Zhao X, Cai G, et al. microRNA-103a functions as a mechanosensitive microRNA to inhibit bone formation through targeting Runx2. J Bone Miner Res. 2015 Feb;30(2):330–45. doi:10.1002/jbmr.2352 PubMed PMID: 25195535.

18.	Chen Z, Zhao F, Liang C, Hu L, Li D, Zhang Y, et al. Silencing of miR-138-5p sensitizes bone anabolic action to mechanical stimuli. Theranostics. 2020;10(26):12263–78. doi:10.7150/thno.53009 PubMed PMID: 33204341; PubMed Central PMCID: PMC7667683.

19.	Zhang X, Li Z, Zhao Z, Chen Y, Sun Y, Cai Q. Runx1/miR-26a/Jagged1 signaling axis controls osteoclastogenesis and alleviates orthodontically induced inflammatory root resorption. Int Immunopharmacol. 2021 Nov;100:107991. doi:10.1016/j.intimp.2021.107991 PubMed PMID: 34438336.

20.	Liu M, Sun F, Feng Y, Sun X, Li J, Fan Q, et al. MicroRNA-132-3p represses Smad5 in MC3T3-E1 osteoblastic cells under cyclic tensile stress. Mol Cell Biochem. 2019 Aug;458(1–2):143–57. doi:10.1007/s11010-019-03538-3 PubMed PMID: 31004309.

21.	Liu L, Liu M, Li R, Liu H, Du L, Chen H, et al. MicroRNA-503-5p inhibits stretch-induced osteogenic differentiation and bone formation. Cell Biol Int. 2017 Feb;41(2):112–23. doi:10.1002/cbin.10704 PubMed PMID: 27862699.

22.	Xu Y, Xin R, Sun H, Long D, Li Z, Liao H, et al. Long Non-coding RNAs LOC100126784 and POM121L9P Derived From Bone Marrow Mesenchymal Stem Cells Enhance Osteogenic Differentiation via the miR-503-5p/SORBS1 Axis. Front Cell Dev Biol. 2021;9:723759. doi:10.3389/fcell.2021.723759 PubMed PMID: 34746123; PubMed Central PMCID: PMC8570085.

23.	Kanzaki H, Wada S, Yamaguchi Y, Katsumata Y, Itohiya K, Fukaya S, et al. Compression and tension variably alter Osteoprotegerin expression via miR-3198 in periodontal ligament cells. BMC Mol Cell Biol. 2019 Apr 4;20(1):6. doi:10.1186/s12860-019-0187-2 PubMed PMID: 31041888; PubMed Central PMCID: PMC6449962.

24.	Wang Y, Zheng Y, Li W. Compression loading of osteoclasts attenuated microRNA-146a-5p expression, which promotes angiogenesis by targeting adiponectin. Sci China Life Sci. 2022 Jan;65(1):151–66. doi:10.1007/s11427-020-1869-7 PubMed PMID: 33677819.

25.	Wang X, Geng B, Wang H, Wang S, Zhao D, He J, et al. Fluid shear stress-induced down-regulation of microRNA-140-5p promotes osteoblast proliferation by targeting VEGFA via the ERK5 pathway. Connect Tissue Res. 2022 Mar;63(2):156–68. doi:10.1080/03008207.2021.1891228 PubMed PMID: 33588662.

26.	Zhang K, Liu X, Tang Y, Liu Z, Yi Q, Wang L, et al. Fluid Shear Stress Promotes Osteoblast Proliferation and Suppresses Mitochondrial-Mediated Osteoblast Apoptosis Through the miR-214-3p-ATF4 Signaling Axis. Physiol Res. 2022 Aug 31;71(4):527–38. doi:10.33549/physiolres.934917 PubMed PMID: 35770472; PubMed Central PMCID: PMC9616584.

27.	Iwawaki Y, Mizusawa N, Iwata T, Higaki N, Goto T, Watanabe M, et al. MiR-494-3p induced by compressive force inhibits cell proliferation in MC3T3-E1 cells. J Biosci Bioeng. 2015 Oct;120(4):456–62. doi:10.1016/j.jbiosc.2015.02.006 PubMed PMID: 25795570.

28.	Zeng Y, Qu X, Li H, Huang S, Wang S, Xu Q, et al. MicroRNA-100 regulates osteogenic differentiation of human adipose-derived mesenchymal stem cells by targeting BMPR2. FEBS Lett. 2012 Jul 30;586(16):2375–81. doi:10.1016/j.febslet.2012.05.049 PubMed PMID: 22684006.

29.	Xue N, Qi L, Zhang G, Zhang Y. miRNA-125b Regulates Osteogenic Differentiation of Periodontal Ligament Cells Through NKIRAS2/NF-κB Pathway. Cell Physiol Biochem. 2018;48(4):1771–81. doi:10.1159/000492350 PubMed PMID: 30078007.

30.	Xu Y, Ren C, Zhao X, Wang W, Zhang N. microRNA-132 inhibits osteogenic differentiation of periodontal ligament stem cells via GDF5 and the NF-κB signaling pathway. Pathol Res Pract. 2019 Dec;215(12):152722. doi:10.1016/j.prp.2019.152722 PubMed PMID: 31718857.

31.	Zhou X, Luan X, Chen Z, Francis M, Gopinathan G, Li W, et al. MicroRNA-138 Inhibits Periodontal Progenitor Differentiation under Inflammatory Conditions. J Dent Res. 2016 Feb;95(2):230–7. doi:10.1177/0022034515613043 PubMed PMID: 26518300; PubMed Central PMCID: PMC4720956.

32.	Xue K, Hu G, Wu L, Han H, Sun Y, Gan K, et al. The bioceramic sealer iRoot SP promotes osteogenic differentiation of human stem cells from apical papilla via miR-141-3p/SPAG9/MAPK signalling pathway. Int Endod J. 2023 Oct;56(10):1241–53. doi:10.1111/iej.13948 PubMed PMID: 37357722.

33.	Sun Y, Shi J, Luo X, Xu X. microRNA-142-3p regulates osteogenic differentiation of human periodontal ligament stem cells via mediating SGK1. J Stomatol Oral Maxillofac Surg. 2023 Feb;124(1S):101369. doi:10.1016/j.jormas.2022.101369 PubMed PMID: 36565809.

34.	Xie YF, Shu R, Jiang SY, Liu DL, Ni J, Zhang XL. MicroRNA-146 inhibits pro-inflammatory cytokine secretion through IL-1 receptor-associated kinase 1 in human gingival fibroblasts. J Inflamm (Lond). 2013 May 16;10(1):20. doi:10.1186/1476-9255-10-20 PubMed PMID: 23680172; PubMed Central PMCID: PMC3660163.

35.	Jiang SY, Xue D, Xie YF, Zhu DW, Dong YY, Wei CC, et al. The negative feedback regulation of microRNA-146a in human periodontal ligament cells after Porphyromonas gingivalis lipopolysaccharide stimulation. Inflamm Res. 2015 Jun;64(6):441–51. doi:10.1007/s00011-015-0824-y PubMed PMID: 25948157.

36.	Nakasa T, Shibuya H, Nagata Y, Niimoto T, Ochi M. The inhibitory effect of microRNA-146a expression on bone destruction in collagen-induced arthritis. Arthritis Rheum. 2011 Jun;63(6):1582–90. doi:10.1002/art.30321 PubMed PMID: 21425254.

37.	Hung PS, Chen FC, Kuang SH, Kao SY, Lin SC, Chang KW. miR-146a induces differentiation of periodontal ligament cells. J Dent Res. 2010 Mar;89(3):252–7. doi:10.1177/0022034509357411 PubMed PMID: 20110513.

38.	Xie Y feng, Shu R, Jiang S yun, Liu D li, Zhang X li. Comparison of microRNA profiles of human periodontal diseased and healthy gingival tissues. Int J Oral Sci. 2011 Jul;3(3):125–34. doi:10.4248/IJOS11046 PubMed PMID: 21789961; PubMed Central PMCID: PMC3470093.

39.	Nakasa T, Miyaki S, Okubo A, Hashimoto M, Nishida K, Ochi M, et al. Expression of microRNA-146 in rheumatoid arthritis synovial tissue. Arthritis Rheum. 2008 May;58(5):1284–92. doi:10.1002/art.23429 PubMed PMID: 18438844; PubMed Central PMCID: PMC2749927.

40.	Matysiak M, Fortak-Michalska M, Szymanska B, Orlowski W, Jurewicz A, Selmaj K. MicroRNA-146a negatively regulates the immunoregulatory activity of bone marrow stem cells by targeting prostaglandin E2 synthase-2. J Immunol. 2013 May 15;190(10):5102–9. doi:10.4049/jimmunol.1202397 PubMed PMID: 23589616.

41.	Zhao S, Cheng Y, Kim JG. microRNA-146a downregulates IL-17 and IL-35 and inhibits proliferation of human periodontal ligament stem cells. J Cell Biochem. 2019 Aug;120(8):13861–6. doi:10.1002/jcb.28659 PubMed PMID: 30989711.

42.	Vimalraj S, Partridge NC, Selvamurugan N. A positive role of microRNA-15b on regulation of osteoblast differentiation. J Cell Physiol. 2014 Sep;229(9):1236–44. doi:10.1002/jcp.24557 PubMed PMID: 24435757; PubMed Central PMCID: PMC4038448.

43.	Wu D, Ma L. Downregulating microRNA-152-3p promotes the viability and osteogenic differentiation of periodontal ligament stem cells via targeting integrin alpha 5. Arch Oral Biol. 2020 Dec;120:104930. doi:10.1016/j.archoralbio.2020.104930 PubMed PMID: 33059275.

44.	Jia J, Feng X, Xu W, Yang S, Zhang Q, Liu X, et al. MiR-17-5p modulates osteoblastic differentiation and cell proliferation by targeting SMAD7 in non-traumatic osteonecrosis. Exp Mol Med. 2014 Jul 25;46(7):e107. doi:10.1038/emm.2014.43 PubMed PMID: 25060766; PubMed Central PMCID: PMC4119212.

45.	Liu Y, Liu W, Hu C, Xue Z, Wang G, Ding B, et al. MiR-17 modulates osteogenic differentiation through a coherent feed-forward loop in mesenchymal stem cells isolated from periodontal ligaments of patients with periodontitis. Stem Cells. 2011 Nov;29(11):1804–16. doi:10.1002/stem.728 PubMed PMID: 21898695.

46.	Jiao Y, Mi S, Li X, Liu Y, Han N, Xu J, et al. MicroRNA-155 targets SOCS1 to inhibit osteoclast differentiation during orthodontic tooth movement. BMC Oral Health. 2023 Dec 1;23(1):955. doi:10.1186/s12903-023-03443-8 PubMed PMID: 38041017; PubMed Central PMCID: PMC10693016.

47.	Li C, Duan G, Feng Y. Downregulation of miR-184 facilitates osseous differentiation in periodontal ligament stem cells by modulating nuclear factor I-C. J Dent Sci. 2021 Mar;16(2):668–75. doi:10.1016/j.jds.2020.09.011 PubMed PMID: 33854717; PubMed Central PMCID: PMC8025194.

48.	Jeong BC, Kang IH, Hwang YC, Kim SH, Koh JT. MicroRNA-194 reciprocally stimulates osteogenesis and inhibits adipogenesis via regulating COUP-TFII expression. Cell Death Dis. 2014 Nov 20;5(11):e1532. doi:10.1038/cddis.2014.485 PubMed PMID: 25412310; PubMed Central PMCID: PMC4260743.

49.	Li J, Han Q, Chen H, Liu T, Song J, Hou M, et al. Carbon Monoxide-Releasing Molecule-3 Enhances Osteogenic Differentiation of Rat Bone Marrow Mesenchymal Stem Cells via miR-195-5p/Wnt3a Pathway. Drug Des Devel Ther. 2022;16:2101–17. doi:10.2147/DDDT.S367277 PubMed PMID: 35812136; PubMed Central PMCID: PMC9259429.

50.	Hu J, Huang X, Zheng L, Zhang Y, Zeng H, Nie L, et al. MiR-199a-5P promotes osteogenic differentiation of human stem cells from apical papilla via targeting IFIT2 in apical periodontitis. Front Immunol. 2023;14:1149339. doi:10.3389/fimmu.2023.1149339 PubMed PMID: 37063854; PubMed Central PMCID: PMC10098181.

51.	Wang Q, Lin H, Ran J, Jiang Z, Ren Q, He W, et al. miR-200a-3p represses osteogenesis of human periodontal ligament stem cells by targeting ZEB2 and activating the NF-κB pathway. Acta Odontol Scand. 2022 Mar;80(2):140–9. doi:10.1080/00016357.2021.1964593 PubMed PMID: 34632930.

52.	Li M, Wei L, Zhou W, He Z, Ran S, Liang J. miR-200a contributes to the migration of BMSCs induced by the secretions of E. faecalis via FOXJ1/NFκB/MMPs axis. Stem Cell Res Ther. 2020 Jul 25;11(1):317. doi:10.1186/s13287-020-01833-1 PubMed PMID: 32711573; PubMed Central PMCID: PMC7382064.

53.	Akkouch A, Eliason S, Sweat ME, Romero-Bustillos M, Zhu M, Qian F, et al. Enhancement of MicroRNA-200c on Osteogenic Differentiation and Bone Regeneration by Targeting Sox2-Mediated Wnt Signaling and Klf4. Hum Gene Ther. 2019 Nov;30(11):1405–18. doi:10.1089/hum.2019.019 PubMed PMID: 31288577; PubMed Central PMCID: PMC6854517.

54.	Strauss FJ, Stähli A, Kobatake R, Tangl S, Heimel P, Apaza Alccayhuaman KA, et al. miRNA-21 deficiency impairs alveolar socket healing in mice. J Periodontol. 2020 Dec;91(12):1664–72. doi:10.1002/JPER.19-0567 PubMed PMID: 32396233; PubMed Central PMCID: PMC7818433.

55.	Wei F, Yang S, Guo Q, Zhang X, Ren D, Lv T, et al. MicroRNA-21 regulates Osteogenic Differentiation of Periodontal Ligament Stem Cells by targeting Smad5. Sci Rep. 2017 Nov 30;7(1):16608. doi:10.1038/s41598-017-16720-8 PubMed PMID: 29192241; PubMed Central PMCID: PMC5709498.

56.	Wu T, Liu Y, Fan Z, Xu J, Jin L, Gao Z, et al. miR-21 Modulates the Immunoregulatory Function of Bone Marrow Mesenchymal Stem Cells Through the PTEN/Akt/TGF-β1 Pathway. Stem Cells. 2015 Nov;33(11):3281–90. doi:10.1002/stem.2081 PubMed PMID: 26086742.

57.	Wu L, Su Y, Lin F, Zhu S, Wang J, Hou Y, et al. MicroRNA-21 promotes orthodontic tooth movement by modulating the RANKL/OPG balance in T cells. Oral Dis. 2020 Mar;26(2):370–80. doi:10.1111/odi.13239 PubMed PMID: 31738470.

58.	Oka S, Li X, Zhang F, Tewari N, Ma R, Zhong L, et al. MicroRNA-21 facilitates osteoblast activity. Biochem Biophys Rep. 2021 Mar;25:100894. doi:10.1016/j.bbrep.2020.100894 PubMed PMID: 33426313; PubMed Central PMCID: PMC7782325.

59.	Chen N, Sui BD, Hu CH, Cao J, Zheng CX, Hou R, et al. microRNA-21 Contributes to Orthodontic Tooth Movement. J Dent Res. 2016 Nov;95(12):1425–33. doi:10.1177/0022034516657043 PubMed PMID: 27422860.

60.	Yang C, Liu X, Zhao K, Zhu Y, Hu B, Zhou Y, et al. miRNA-21 promotes osteogenesis via the PTEN/PI3K/Akt/HIF-1α pathway and enhances bone regeneration in critical size defects. Stem Cell Res Ther. 2019 Feb 22;10(1):65. doi:10.1186/s13287-019-1168-2 PubMed PMID: 30795815; PubMed Central PMCID: PMC6387542.

61.	Pizzicannella J, Cavalcanti M, Trubiani O, Diomede F. MicroRNA 210 Mediates VEGF Upregulation in Human Periodontal Ligament Stem Cells Cultured on 3DHydroxyapatite Ceramic Scaffold. Int J Mol Sci. 2018 Dec 6;19(12):3916. doi:10.3390/ijms19123916 PubMed PMID: 30563289; PubMed Central PMCID: PMC6320762.

62.	Cao F, Zhan J, Chen X, Zhang K, Lai R, Feng Z. miR-214 promotes periodontal ligament stem cell osteoblastic differentiation by modulating Wnt/β‑catenin signaling. Mol Med Rep. 2017 Dec;16(6):9301–8. doi:10.3892/mmr.2017.7821 PubMed PMID: 29152645; PubMed Central PMCID: PMC5779983.

63.	Yan GQ, Wang X, Yang F, Yang ML, Zhang GR, Wang GK, et al. MicroRNA-22 Promoted Osteogenic Differentiation of Human Periodontal Ligament Stem Cells by Targeting HDAC6. J Cell Biochem. 2017 Jul;118(7):1653–8. doi:10.1002/jcb.25931 PubMed PMID: 28195408.

64.	Zhang Z, Wang M, Zheng Y, Dai Y, Chou J, Bian X, et al. MicroRNA-223 negatively regulates the osteogenic differentiation of periodontal ligament derived cells by directly targeting growth factor receptors. J Transl Med. 2022 Oct 11;20(1):465. doi:10.1186/s12967-022-03676-1 PubMed PMID: 36221121; PubMed Central PMCID: PMC9552407.

65.	Zhang Y, Li S, Yuan S, Zhang H, Liu J. MicroRNA-23a inhibits osteogenesis of periodontal mesenchymal stem cells by targeting bone morphogenetic protein signaling. Arch Oral Biol. 2019 Jun;102:93–100. doi:10.1016/j.archoralbio.2019.04.001 PubMed PMID: 30981077.

66.	Sun X, Li M, Ban J, Li Z. miR-23b mediates TNF-α-Inhibited Osteogenic Differentiation of Human Periodontal Ligament Stem Cells by Targeting Runx2. Int J Med Sci. 2021;18(16):3674–83. doi:10.7150/ijms.64312 PubMed PMID: 34790039; PubMed Central PMCID: PMC8579284.

67.	Li Z, Sun Y, Cao S, Zhang J, Wei J. Downregulation of miR-24-3p promotes osteogenic differentiation of human periodontal ligament stem cells by targeting SMAD family member 5. J Cell Physiol. 2019 May;234(5):7411–9. doi:10.1002/jcp.27499 PubMed PMID: 30378100.

68.	Ding Y, Luan J, Malmstrom HS, Luan X, Diekwisch TGH. Mir-27 Promotes Periodontal Regeneration via Osteogenesis/Angiogenesis. J Dent Res. 2026 Apr;105(4):495–504. doi:10.1177/00220345251366279 PubMed PMID: 41102998.

69.	Park MG, Kim JS, Park SY, Lee SA, Kim HJ, Kim CS, et al. MicroRNA-27 promotes the differentiation of odontoblastic cell by targeting APC and activating Wnt/β-catenin signaling. Gene. 2014 Apr 1;538(2):266–72. doi:10.1016/j.gene.2014.01.045 PubMed PMID: 24487055.

70.	Kapinas K, Kessler C, Ricks T, Gronowicz G, Delany AM. miR-29 modulates Wnt signaling in human osteoblasts through a positive feedback loop. J Biol Chem. 2010 Aug 13;285(33):25221–31. doi:10.1074/jbc.M110.116137 PubMed PMID: 20551325; PubMed Central PMCID: PMC2919085.

71.	Franceschetti T, Kessler CB, Lee SK, Delany AM. miR-29 promotes murine osteoclastogenesis by regulating osteoclast commitment and migration. J Biol Chem. 2013 Nov 15;288(46):33347–60. doi:10.1074/jbc.M113.484568 PubMed PMID: 24085298; PubMed Central PMCID: PMC3829182.

72.	Baglìo SR, Devescovi V, Granchi D, Baldini N. MicroRNA expression profiling of human bone marrow mesenchymal stem cells during osteogenic differentiation reveals Osterix regulation by miR-31. Gene. 2013 Sep 15;527(1):321–31. doi:10.1016/j.gene.2013.06.021 PubMed PMID: 23827457.

73.	Deng Y, Wu S, Zhou H, Bi X, Wang Y, Hu Y, et al. Effects of a miR-31, Runx2, and Satb2 regulatory loop on the osteogenic differentiation of bone mesenchymal stem cells. Stem Cells Dev. 2013 Aug 15;22(16):2278–86. doi:10.1089/scd.2012.0686 PubMed PMID: 23517179.

74.	Chen L, Holmstrøm K, Qiu W, Ditzel N, Shi K, Hokland L, et al. MicroRNA-34a inhibits osteoblast differentiation and in vivo bone formation of human stromal stem cells. Stem Cells. 2014 Apr;32(4):902–12. doi:10.1002/stem.1615 PubMed PMID: 24307639.

75.	Wang YH, Li SY, Yuan SJ, Pan YX, Hua Y, Liu JY. MiR-375 promotes human periodontal ligament stem cells proliferation and osteogenic differentiation by targeting transducer of ERBB2, 2. Arch Oral Biol. 2020 Sep;117:104818. doi:10.1016/j.archoralbio.2020.104818 PubMed PMID: 32619704.

76.	Ma L, Wu D. MicroRNA-383-5p regulates osteogenic differentiation of human periodontal ligament stem cells by targeting histone deacetylase 9. Arch Oral Biol. 2021 Sep;129:105166. doi:10.1016/j.archoralbio.2021.105166 PubMed PMID: 34118749.

77.	Shi F, He R, Zhu J, Lu T, Zhong L. miR-589-3p promoted osteogenic differentiation of periodontal ligament stem cells through targeting ATF1. J Orthop Surg Res. 2022 Apr 10;17(1):221. doi:10.1186/s13018-022-03000-z PubMed PMID: 35399081; PubMed Central PMCID: PMC8996605.

78.	Iwata T, Mizuno N, Nagahara T, Kaneda-Ikeda E, Kajiya M, Sasaki S, et al. Cytokines regulate stemness of mesenchymal stem cells via miR-628-5p during periodontal regeneration. J Periodontol. 2022 Feb;93(2):269–86. doi:10.1002/JPER.21-0064 PubMed PMID: 34152611.

79.	Zhang HS, Jiang CX, Ji YT, Zhang YF, Chen Z, Cao ZG, et al. Osteoprotective Role of the Mir338 Cluster Ablation during Periodontitis. J Dent Res. 2023 Nov;102(12):1337–47. doi:10.1177/00220345231187288 PubMed PMID: 37688381.

80.	Guo J, Ouyang XY, Liu JR, Liu WY, Wang YB. miR-508-5p suppresses osteogenic differentiation of human periodontal ligament stem cells via targeting sex-determining region Y-related HMG-box 11. J Dent Sci. 2025 Jan;20(1):201–11. doi:10.1016/j.jds.2024.08.012 PubMed PMID: 39873049; PubMed Central PMCID: PMC11763229.

81.	Mi J, Wang S, Liu P, Liu C, Zhuang D, Leng X, et al. CUL4B Upregulates RUNX2 to Promote the Osteogenic Differentiation of Human Periodontal Ligament Stem Cells by Epigenetically Repressing the Expression of miR-320c and miR-372/373-3p. Front Cell Dev Biol. 2022;10:921663. doi:10.3389/fcell.2022.921663 PubMed PMID: 35784474; PubMed Central PMCID: PMC9243338.

82.	Wang C, Miao X, Li Y, Ren L, Zheng B, Jiang Z, et al. Mechanistic insights into miR-584-5p-mediated Inhibition of PDLSCs osteogenic differentiation through H2AFZ upregulation and RUNX2 suppression. Cell Mol Life Sci. 2025 Oct 7;82(1):351. doi:10.1007/s00018-025-05887-3 PubMed PMID: 41055656; PubMed Central PMCID: PMC12504162.

83.	Yang S, Gao J, Chen M, Sun Y, Qiao X, Mao H, et al. Let-7a promotes periodontal bone regeneration of bone marrow mesenchymal stem cell aggregates via the Fas/FasL-autophagy pathway. J Cell Mol Med. 2023 Dec;27(24):4056–68. doi:10.1111/jcmm.17988 PubMed PMID: 37855249; PubMed Central PMCID: PMC10746947.

84.	Benakanakere MR, Li Q, Eskan MA, Singh AV, Zhao J, Galicia JC, et al. Modulation of TLR2 protein expression by miR-105 in human oral keratinocytes. J Biol Chem. 2009 Aug 21;284(34):23107–15. doi:10.1074/jbc.M109.013862 PubMed PMID: 19509287; PubMed Central PMCID: PMC2755716.

85.	Na HS, Park MH, Song YR, Kim S, Kim HJ, Lee JY, et al. Elevated MicroRNA-128 in Periodontitis Mitigates Tumor Necrosis Factor-α Response via p38 Signaling Pathway in Macrophages. J Periodontol. 2016 Sep;87(9):e173-182. doi:10.1902/jop.2016.160033 PubMed PMID: 27240473.

86.	Wu L, Zhang G, Guo C, Zhao X, Shen D, Yang N. MiR-128-3p mediates TNF-α-induced inflammatory responses by regulating Sirt1 expression in bone marrow mesenchymal stem cells. Biochem Biophys Res Commun. 2020 Jan 1;521(1):98–105. doi:10.1016/j.bbrc.2019.10.083 PubMed PMID: 31635801.

87.	Wu S, Wang J, Yang D. IL-6/STAT3-Mediated miR-181a-5p in Bone Marrow-Derived Mesenchymal Stem Cells Regulates Th17/Treg Balance in Experimental Periodontitis. Int Dent J. 2026 Aug;76(4):109675. doi:10.1016/j.identj.2026.109675 PubMed PMID: 42275747; PubMed Central PMCID: PMC13276558.

88.	Hua B, Xiang J, Guo L, Lu D. MicroRNA-212-5p regulates the inflammatory response of periodontal ligament cells by targeting myeloid differentiation factor 88. Arch Oral Biol. 2020 Oct;118:104831. doi:10.1016/j.archoralbio.2020.104831 PubMed PMID: 32707140.

89.	Chaparro A, Lozano M, Gaedechens D, López C, Albers D, Hernández M, et al. Exploring the Expression of Pro-Inflammatory and Hypoxia-Related MicroRNA-20a, MicroRNA-30e, and MicroRNA-93 in Periodontitis and Gingival Mesenchymal Stem Cells under Hypoxia. Int J Mol Sci. 2022 Sep 7;23(18):10310. doi:10.3390/ijms231810310 PubMed PMID: 36142220; PubMed Central PMCID: PMC9499533.

90.	Moffatt CE, Lamont RJ. Porphyromonas gingivalis induction of microRNA-203 expression controls suppressor of cytokine signaling 3 in gingival epithelial cells. Infect Immun. 2011 Jul;79(7):2632–7. doi:10.1128/IAI.00082-11 PubMed PMID: 21536793; PubMed Central PMCID: PMC3191996.

91.	P H, L J. MicroRNA-28-5p as a potential diagnostic biomarker for chronic periodontitis and its role in cell proliferation and inflammatory response. Journal of dental sciences. 2022 Oct;17(4). doi:10.1016/j.jds.2022.04.031 PubMed PMID: 36299340.

92.	Du A, Zhao S, Wan L, Liu T, Peng Z, Zhou Z, et al. MicroRNA expression profile of human periodontal ligament cells under the influence of Porphyromonas gingivalis LPS. J Cell Mol Med. 2016 Jul;20(7):1329–38. doi:10.1111/jcmm.12819 PubMed PMID: 26987780; PubMed Central PMCID: PMC4929301.

93.	Wu Y, Ji Y, Mu R, Pan J, Fan R, Zhao Y, et al. The Biological Role of the Signal Transducer and Activator of Transcription 1 (STAT1)-miR-221/222-3p-p21 Activated Kinase 1 (PAK1) Axis in Experimental Periodontitis. Inflammation. 2025 Dec;48(6):4110–25. doi:10.1007/s10753-025-02314-4 PubMed PMID: 40423866; PubMed Central PMCID: PMC12722484.

94.	Deng L, Huo PC, Feng MT, Wang RL, Jing R, Luo LJ. miR-27a-5p alleviates periodontal inflammation by targeting phosphatase and tensin homolog deleted on chromosome ten. Mol Oral Microbiol. 2023 Aug;38(4):309–20. doi:10.1111/omi.12416 PubMed PMID: 37216657.

95.	Li W, Wang J, Hao W, Yu C. MicroRNA-543-3p down-regulates inflammation and inhibits periodontitis through KLF6. Biosci Rep. 2021 May 28;41(5):BSR20210138. doi:10.1042/BSR20210138 PubMed PMID: 33955459; PubMed Central PMCID: PMC8144941.

96.	Ouhara K, Savitri IJ, Fujita T, Kittaka M, Kajiya M, Iwata T, et al. miR-584 expressed in human gingival epithelial cells is induced by Porphyromonas gingivalis stimulation and regulates interleukin-8 production via lactoferrin receptor. J Periodontol. 2014 Jun;85(6):e198-204. doi:10.1902/jop.2013.130335 PubMed PMID: 24228808.

97.	Wang S, Ren Y, Li J, Li H, Li J, Lan X, et al. MicroRNA-671-5p regulates the inflammatory response of periodontal ligament stem cells via the DUSP8/p38 MAPK pathway. Mol Biol Rep. 2024 May 10;51(1):644. doi:10.1007/s11033-024-09510-9 PubMed PMID: 38727958.

98.	Zhong X, Wang H. circSKIL promotes osteoblastic differentiation of periodontal ligament cells by sponging miR-532-5p to activate Notch signaling. J Periodontal Res. 2022 Dec;57(6):1148–58. doi:10.1111/jre.13052 PubMed PMID: 36063416.

99.	Ye Y, Ke Y, Liu L, Xiao T, Yu J. CircRNA FAT1 Regulates Osteoblastic Differentiation of Periodontal Ligament Stem Cells via miR-4781-3p/SMAD5 Pathway. Stem Cells Int. 2021;2021:5177488. doi:10.1155/2021/5177488 PubMed PMID: 35003269; PubMed Central PMCID: PMC8731273.

100.	Li X, Zheng Y, Zheng Y, Huang Y, Zhang Y, Jia L, et al. Circular RNA CDR1as regulates osteoblastic differentiation of periodontal ligament stem cells via the miR-7/GDF5/SMAD and p38 MAPK signaling pathway. Stem Cell Res Ther. 2018 Aug 31;9(1):232. doi:10.1186/s13287-018-0976-0 PubMed PMID: 30170617; PubMed Central PMCID: PMC6119336.

101.	Xiao T, Shi Y, Ye Y, Wang J, Wang W, Yu H, et al. Circ-SPATA13 regulates the osteogenic differentiation of human periodontal ligament stem cells through the miR-485-5p_R + 1/BMP7 axis. Cell Signal. 2025 Mar;127:111561. doi:10.1016/j.cellsig.2024.111561 PubMed PMID: 39667547.

102.	Zhao X, Sun W, Guo B, Cui L. Circular RNA BIRC6 depletion promotes osteogenic differentiation of periodontal ligament stem cells via the miR-543/PTEN/PI3K/AKT/mTOR signaling pathway in the inflammatory microenvironment. Stem Cell Res Ther. 2022 Aug 13;13(1):417. doi:10.1186/s13287-022-03093-7 PubMed PMID: 35964136; PubMed Central PMCID: PMC9375426.

103.	Wang L, Liu Y, Diao Z, Huang Y, Yang H, Zhang Y, et al. The circ_0054633/miR-590-3p/RUNX2 positive feedback loop promotes the osteogenic differentiation of BMSCs. Stem Cell Res Ther. 2025 Jul 1;16(1):325. doi:10.1186/s13287-025-04450-y PubMed PMID: 40597390; PubMed Central PMCID: PMC12210457.

104.	Wang K, Zhang C, Mai Y, Zhang Y, Li Y, Li C. mmu_circ_0000217 Promotes Osteogenic Differentiation in OCCM-30 Cells via the miR-3064-3p/DKK1 Axis. Adv Biol (Weinh). 2026 Jan;10(1):e00581. doi:10.1002/adbi.202500581 PubMed PMID: 41589913.

105.	Qi Y, Lin C, Zhao C, Wu Y. Circ_0003072 Mediates the Pro-osteogenic Differentiation Effect of Betulinic Acid on Human Periodontal Ligament Stem Cells. Int Dent J. 2025 Apr;75(2):1390–9. doi:10.1016/j.identj.2024.10.017 PubMed PMID: 39572284; PubMed Central PMCID: PMC11976600.

106.	Zhou J, Sui M, Ji F, Shen S, Lin Y, Jin M, et al. Hsa_circ_0036872 has an important promotional effect in enhancing osteogenesis of dental pulp stem cells by regulating the miR-143-3p/IGF2 axis. Int Immunopharmacol. 2024 Mar 30;130:111744. doi:10.1016/j.intimp.2024.111744 PubMed PMID: 38412676.

107.	Ren L, He L, Zhang R, Li X, Xiao S, Guo S, et al. Hsa_circ_0007349 upregulates glutamate-ammonia ligase to modulate gingival fibroblast wound healing. Arch Oral Biol. 2025 Dec;180:106419. doi:10.1016/j.archoralbio.2025.106419 PubMed PMID: 41075359.

108.	Huang Y, Han Y, Guo R, Liu H, Li X, Jia L, et al. Long non-coding RNA FER1L4 promotes osteogenic differentiation of human periodontal ligament stromal cells via miR-874-3p and vascular endothelial growth factor A. Stem Cell Res Ther. 2020 Jan 3;11(1):5. doi:10.1186/s13287-019-1519-z PubMed PMID: 31900200; PubMed Central PMCID: PMC6942378.

109.	Zhong X, Wang H. LncRNA JHDM1D-AS1 promotes osteogenic differentiation of periodontal ligament cells by targeting miR-532-5p to activate IGF1R signaling. J Periodontal Res. 2024 Feb;59(1):220–30. doi:10.1111/jre.13209 PubMed PMID: 37950511.

110.	Xiang J, Bian Y. PWAR6 interacts with miR‑106a‑5p to regulate the osteogenic differentiation of human periodontal ligament stem cells. Mol Med Rep. 2021 Apr;23(4):268. doi:10.3892/mmr.2021.11907 PubMed PMID: 33576453; PubMed Central PMCID: PMC7893692.

111.	Liu Y, Liu C, Zhang A, Yin S, Wang T, Wang Y, et al. Down-regulation of long non-coding RNA MEG3 suppresses osteogenic differentiation of periodontal ligament stem cells (PDLSCs) through miR-27a-3p/IGF1 axis in periodontitis. Aging (Albany NY). 2019 Aug 9;11(15):5334–50. doi:10.18632/aging.102105 PubMed PMID: 31398715; PubMed Central PMCID: PMC6710065.

112.	Ye C, Ye J, Wu H, Zhang F, Liu Y. Role of maternally expressed 8 small nucleolar RNA (MEG8) in osteogenic differentiation of periodontal ligament stem cells. Oral Dis. 2024 Jul;30(5):3351–62. doi:10.1111/odi.14759 PubMed PMID: 38155397.

113.	Sun X, Li Z, Dong S, Dong Q. Long non‑coding RNA SNHG5 promotes osteogenic differentiation of human periodontal ligament stem cells via mediating miR‑23b‑3p/Runx2 axis. Int J Med Sci. 2023;20(7):958–68. doi:10.7150/ijms.82454 PubMed PMID: 37324192; PubMed Central PMCID: PMC10266046.

114.	Li X, Huang Y, Han Y, Yang Q, Zheng Y, Li W. LncPVT1 regulates osteogenic differentiation of human periodontal ligament cells via miR-10a-5p/brain-derived neurotrophic factor. J Periodontol. 2022 Jul;93(7):1093–106. doi:10.1002/JPER.21-0429 PubMed PMID: 34793611.

115.	Hua L, Zhang X. MALAT1 regulates osteogenic differentiation of human periodontal ligament stem cells through mediating miR-155-5p/ETS1 axis. Tissue Cell. 2021 Dec;73:101619. doi:10.1016/j.tice.2021.101619 PubMed PMID: 34399118.

116.	Gu Y, Bai Y. LncRNA MALAT1 promotes osteogenic differentiation through the miR-93-5p/SMAD5 axis. Oral Dis. 2024 May;30(4):2398–409. doi:10.1111/odi.14705 PubMed PMID: 37533355.

117.	Gu N, Wang Y, Li L, Sui X, Liu Z. The mechanism of lncRNA MALAT1 targeting the miR-124-3p/IGF2BP1 axis to regulate osteogenic differentiation of periodontal ligament stem cells. Clin Oral Investig. 2024 Mar 16;28(4):219. doi:10.1007/s00784-024-05616-3 PubMed PMID: 38492123.

118.	Ahmad I, Naqvi RA, Valverde A, Naqvi AR. LncRNA MALAT1/microRNA-30b axis regulates macrophage polarization and function. Front Immunol. 2023;14:1214810. doi:10.3389/fimmu.2023.1214810 PubMed PMID: 37860007; PubMed Central PMCID: PMC10582718.

119.	Guo J, Zheng M. The regulation mechanism of LINC00707 on the osteogenic differentiation of human periodontal ligament stem cells. J Mol Histol. 2022 Feb;53(1):13–26. doi:10.1007/s10735-021-10029-7 PubMed PMID: 34674104.

120.	Zhai S, Liu C, Vimalraj S, Subramanian R, Abullais SS, Arora S, et al. Glucagon-like peptide-1 receptor promotes osteoblast differentiation of dental pulp stem cells and bone formation in a zebrafish scale regeneration model. Peptides. 2023 May;163:170974. doi:10.1016/j.peptides.2023.170974 PubMed PMID: 36775021.

121.	Liao C, Zhou Y, Li M, Xia Y, Peng W. LINC00968 promotes osteogenic differentiation in vitro and bone formation in vivo via regulation of miR-3658/RUNX2. Differentiation. 2020;116:1–8. doi:10.1016/j.diff.2020.09.005 PubMed PMID: 33065511.

122.	Lin C, Liu M, Guo J, Jia R. Cryotherapy Attenuates Inflammation via the lncRNA SNHG1/miR-9-5p/NFKB1 Regulatory Axis in Periodontal Ligament Cells. Int J Mol Sci. 2023 Jul 28;24(15):12097. doi:10.3390/ijms241512097 PubMed PMID: 37569472; PubMed Central PMCID: PMC10418934.

123.	Zhang X, Wang Y, Zhao H, Han X, Zhao T, Qu P, et al. Extracellular vesicle-encapsulated miR-22-3p from bone marrow mesenchymal stem cell promotes osteogenic differentiation via FTO inhibition. Stem Cell Res Ther. 2020 Jun 10;11(1):227. doi:10.1186/s13287-020-01707-6 PubMed PMID: 32522250; PubMed Central PMCID: PMC7285613.

124.	Qu F, Zhang YF, Wang YY, Cao XM, Shen YY, Wu ZA, et al. Cyclic stretch-induced exosomes from periodontal ligament cells promote osteoblasts osteogenic differentiation via the miR-181d-5p/TNF signaling pathway. Arch Oral Biol. 2024 Jan;157:105843. doi:10.1016/j.archoralbio.2023.105843 PubMed PMID: 37939516.

125.	Lai S, Deng L, Liu C, Li X, Fan L, Zhu Y, et al. Bone marrow mesenchymal stem cell-derived exosomes loaded with miR-26a through the novel immunomodulatory peptide DP7-C can promote osteogenesis. Biotechnol Lett. 2023 Jul;45(7):905–19. doi:10.1007/s10529-023-03376-w PubMed PMID: 37195490.

126.	Xu XY, Tian BM, Xia Y, Xia YL, Li X, Zhou H, et al. Exosomes derived from P2X7 receptor gene-modified cells rescue inflammation-compromised periodontal ligament stem cells from dysfunction. Stem Cells Transl Med. 2020 Nov;9(11):1414–30. doi:10.1002/sctm.19-0418 PubMed PMID: 32597574; PubMed Central PMCID: PMC7581448.

127.	Liu T, Hu W, Zou X, Xu J, He S, Chang L, et al. Human Periodontal Ligament Stem Cell-Derived Exosomes Promote Bone Regeneration by Altering MicroRNA Profiles. Stem Cells Int. 2020;2020:8852307. doi:10.1155/2020/8852307 PubMed PMID: 33293963; PubMed Central PMCID: PMC7691010.

128.	Yan C, Li N, Xiao T, Ye X, Fu L, Ye Y, et al. Extracellular vesicles from the inflammatory microenvironment regulate the osteogenic and odontogenic differentiation of periodontal ligament stem cells by miR-758-5p/LMBR1/BMP2/4 axis. J Transl Med. 2022 May 13;20(1):208. doi:10.1186/s12967-022-03412-9 PubMed PMID: 35562763; PubMed Central PMCID: PMC9103284.

129.	Lai S, Tang N, Guo J, Deng L, Yuan L, Zeng L, et al. Immunomodulatory peptide DP7-C mediates macrophage-derived exosomal miR-21b to promote bone regeneration via the SOCS1/JAK2/STAT3 axis. Colloids Surf B Biointerfaces. 2025 Sep;253:114709. doi:10.1016/j.colsurfb.2025.114709 PubMed PMID: 40286607.

130.	Zhang Y, Liang Y, Zhou Y. RAW 264.7-derived exosomal miR-494-3p regulates inflammation and osteogenic differentiation of human periodontal ligament stem cells through regulating CAMK2D. Arch Oral Biol. 2026 Feb;182:106457. doi:10.1016/j.archoralbio.2025.106457 PubMed PMID: 41240683.

131.	Gao Y, Li X, Ding Y, Wang Y, Du J, Chen Y, et al. MiR-451a-Enriched Small Extracellular Vesicles Derived from Mg2+-Activated DPSCs Induce Vascularized Bone Regeneration through the AKT/eNOS/NO Axis. ACS Appl Mater Interfaces. 2025 May 28;17(21):31345–56. doi:10.1021/acsami.5c02551 PubMed PMID: 40364481.

132.	Liu S, Wang Z, Li Y, Pan Z, Huang L, Cui J, et al. Erythropoietin-Stimulated Macrophage-Derived Extracellular Vesicles in Chitosan Hydrogel Rescue BMSCs Fate by Targeting EGFR to Alleviate Inflammatory Bone Loss in Periodontitis. Adv Sci (Weinh). 2025 Jun;12(23):e2500554. doi:10.1002/advs.202500554 PubMed PMID: 40289904; PubMed Central PMCID: PMC12199399.

133.	Chen L, Zhang J, Yu J, Guo S, Tian W. LPS pretreated dental follicle stem cell derived exosomes promote periodontal tissue regeneration via miR-184 and PPARα-Akt-JNK signaling pathway. Stem Cell Res Ther. 2025 Jul 2;16(1):347. doi:10.1186/s13287-025-04462-8 PubMed PMID: 40605003; PubMed Central PMCID: PMC12224499.

134.	Zhuang XM, Zhou B. Exosome secreted by human gingival fibroblasts in radiation therapy inhibits osteogenic differentiation of bone mesenchymal stem cells by transferring miR-23a. Biomed Pharmacother. 2020 Nov;131:110672. doi:10.1016/j.biopha.2020.110672 PubMed PMID: 32889404.

135.	Lin C, Yang Y, Wang Y, Jing H, Bai X, Hong Z, et al. Periodontal ligament fibroblasts-derived exosomes induced by PGE2 inhibit human periodontal ligament stem cells osteogenic differentiation via activating miR-34c-5p/SATB2/ERK. Exp Cell Res. 2022 Oct 15;419(2):113318. doi:10.1016/j.yexcr.2022.113318 PubMed PMID: 35981635.

136.	Wei Y, Ma H, Zhou H, Yin H, Yang J, Song Y, et al. miR-424-5p shuttled by bone marrow stem cells-derived exosomes attenuates osteogenesis via regulating WIF1-mediated Wnt/β-catenin axis. Aging (Albany NY). 2021 Jul 6;13(13):17190–201. doi:10.18632/aging.203169 PubMed PMID: 34229300; PubMed Central PMCID: PMC8312462.

137.	Liu X, Guo L, Du J, Luo Z, Xu J, Bhawal UK, et al. Macrophage-derived apoptotic bodies impair the osteogenic ability of osteoblasts in periodontitis. Oral Dis. 2024 Jul;30(5):3296–307. doi:10.1111/odi.14808 PubMed PMID: 37994174.

138.	Myint YYK, Liu A, Gao J, Hatasa M, Srithanyarat SS, Iwata T. hsa-let-7c-3p Restrains the Proliferation-to-Osteogenesis Transition of Human Periodontal Ligament Mesenchymal Stem/Stromal Cells via NT5E. Stem Cell Rev Rep. 2026 Aug;22(6):2863–78. doi:10.1007/s12015-026-11160-2 PubMed PMID: 42230452; PubMed Central PMCID: PMC13354647.

139.	Nakao Y, Fukuda T, Zhang Q, Sanui T, Shinjo T, Kou X, et al. Exosomes from TNF-α-treated human gingiva-derived MSCs enhance M2 macrophage polarization and inhibit periodontal bone loss. Acta Biomater. 2021 Mar 1;122:306–24. doi:10.1016/j.actbio.2020.12.046 PubMed PMID: 33359765; PubMed Central PMCID: PMC7897289.

140.	Hayashi C, Fukuda T, Kawakami K, Toyoda M, Nakao Y, Watanabe Y, et al. miR-1260b inhibits periodontal bone loss by targeting ATF6β mediated regulation of ER stress. Front Cell Dev Biol. 2022;10:1061216. doi:10.3389/fcell.2022.1061216 PubMed PMID: 36531939; PubMed Central PMCID: PMC9748617.

141.	Lu J, Yu N, Liu Q, Xie Y, Zhen L. Periodontal Ligament Stem Cell Exosomes Key to Regulate Periodontal Regeneration by miR-31-5p in Mice Model. Int J Nanomedicine. 2023;18:5327–42. doi:10.2147/IJN.S409664 PubMed PMID: 37746047; PubMed Central PMCID: PMC10516219.

142.	Li X, Jiang Y, Liu X, Fu J, Du J, Luo Z, et al. Mesenchymal stem cell-derived apoptotic bodies alleviate alveolar bone destruction by regulating osteoclast differentiation and function. Int J Oral Sci. 2023 Dec 1;15(1):51. doi:10.1038/s41368-023-00255-y PubMed PMID: 38040672; PubMed Central PMCID: PMC10692139.

143.	Xia Y, Cheng T, Zhang C, Zhou M, Hu Z, Kang F, et al. Human bone marrow mesenchymal stem cell-derived extracellular vesicles restore Th17/Treg homeostasis in periodontitis via miR-1246. FASEB J. 2023 Nov;37(11):e23226. doi:10.1096/fj.202300674RR PubMed PMID: 37815505.

144.	Zhang Y, Chen J, Fu H, Kuang S, He F, Zhang M, et al. Exosomes derived from 3D-cultured MSCs improve therapeutic effects in periodontitis and experimental colitis and restore the Th17 cell/Treg balance in inflamed periodontium. Int J Oral Sci. 2021 Dec 14;13(1):43. doi:10.1038/s41368-021-00150-4 PubMed PMID: 34907166; PubMed Central PMCID: PMC8671433.

145.	Shen Z, Kuang S, Zhang Y, Yang M, Qin W, Shi X, et al. Chitosan hydrogel incorporated with dental pulp stem cell-derived exosomes alleviates periodontitis in mice via a macrophage-dependent mechanism. Bioact Mater. 2020 Dec;5(4):1113–26. doi:10.1016/j.bioactmat.2020.07.002 PubMed PMID: 32743122; PubMed Central PMCID: PMC7371600.

146.	Wang Y, Zhang X, Wang J, Zhang Y, Ye Q, Wang Y, et al. Inflammatory Periodontal Ligament Stem Cells Drive M1 Macrophage Polarization via Exosomal miR-143-3p-Mediated Regulation of PI3K/AKT/NF-κB Signaling. Stem Cells. 2023 Mar 2;41(2):184–99. doi:10.1093/stmcls/sxac087 PubMed PMID: 36520505.

147.	Xiao J, Deng Y, Xie J, Liu H, Yang Q, Zhang Y, et al. Apoptotic vesicles from macrophages exacerbate periodontal bone resorption in periodontitis via delivering miR-143-3p targeting Igfbp5. J Nanobiotechnology. 2024 Oct 26;22(1):658. doi:10.1186/s12951-024-02934-2 PubMed PMID: 39456001; PubMed Central PMCID: PMC11515254.

148.	Zheng Y, Dong C, Yang J, Jin Y, Zheng W, Zhou Q, et al. Exosomal microRNA-155-5p from PDLSCs regulated Th17/Treg balance by targeting sirtuin-1 in chronic periodontitis. J Cell Physiol. 2019 Nov;234(11):20662–74. doi:10.1002/jcp.28671 PubMed PMID: 31016751.

149.	Kang L, Miao Y, Jin Y, Shen S, Lin X. Exosomal miR-205-5p derived from periodontal ligament stem cells attenuates the inflammation of chronic periodontitis via targeting XBP1. Immun Inflamm Dis. 2023 Jan;11(1):e743. doi:10.1002/iid3.743 PubMed PMID: 36705422; PubMed Central PMCID: PMC9761342.

150.	Cui S, Zhang Z, Cheng C, Tang S, Zhai M, Li L, et al. Small Extracellular Vesicles from Periodontal Ligament Stem Cells Primed by Lipopolysaccharide Regulate Macrophage M1 Polarization via miR-433-3p Targeting TLR2/TLR4/NF-κB. Inflammation. 2023 Oct;46(5):1849–58. doi:10.1007/s10753-023-01845-y PubMed PMID: 37351818; PubMed Central PMCID: PMC10567992.

151.	Luo P, Jiang C, Ji P, Wang M, Xu J. Exosomes of stem cells from human exfoliated deciduous teeth as an anti-inflammatory agent in temporomandibular joint chondrocytes via miR-100-5p/mTOR. Stem Cell Res Ther. 2019 Jul 29;10(1):216. doi:10.1186/s13287-019-1341-7 PubMed PMID: 31358056; PubMed Central PMCID: PMC6664713.

152.	Wu Y, Qu F, Zhang Y, Song Y, Zhong Q, Huang Y, et al. Exosomes from Cyclic Stretched Periodontal Ligament Cells Induced Periodontal Inflammation through miR-9-5p/SIRT1/NF-κB Signaling Pathway. J Immunol. 2023 Jun 15;210(12):2001–15. doi:10.4049/jimmunol.2300074 PubMed PMID: 37154707.

153.	Yu T, Mi N, Song Y, Xie W. Exosomes miR-92a-3p from human exfoliated deciduous teeth inhibits periodontitis progression via the KLF4/PI3K/AKT pathway. J Periodontal Res. 2024 Aug;59(4):771–82. doi:10.1111/jre.13262 PubMed PMID: 38616305.

154.	Zhou H, Li X, Wu RX, He XT, An Y, Xu XY, et al. Periodontitis-compromised dental pulp stem cells secrete extracellular vesicles carrying miRNA-378a promote local angiogenesis by targeting Sufu to activate the Hedgehog/Gli1 signalling. Cell Prolif. 2021 May;54(5):e13026. doi:10.1111/cpr.13026 PubMed PMID: 33759282; PubMed Central PMCID: PMC8088471.

155.	Liang X, Zhang L, Wang S, Han Q, Zhao RC. Exosomes secreted by mesenchymal stem cells promote endothelial cell angiogenesis by transferring miR-125a. J Cell Sci. 2016 Jun 1;129(11):2182–9. doi:10.1242/jcs.170373 PubMed PMID: 27252357.

156.	Xu F, Xiang Q, Huang J, Chen Q, Yu N, Long X, et al. Exosomal miR-423-5p mediates the proangiogenic activity of human adipose-derived stem cells by targeting Sufu. Stem Cell Res Ther. 2019 Mar 21;10(1):106. doi:10.1186/s13287-019-1196-y PubMed PMID: 30898155; PubMed Central PMCID: PMC6429803.

157.	Zhang Z, Shuai Y, Zhou F, Yin J, Hu J, Guo S, et al. PDLSCs Regulate Angiogenesis of Periodontal Ligaments via VEGF Transferred by Exosomes in Periodontitis. Int J Med Sci. 2020;17(5):558–67. doi:10.7150/ijms.40918 PubMed PMID: 32210705; PubMed Central PMCID: PMC7085218.

158.	Hu X, Zhong Y, Kong Y, Chen Y, Feng J, Zheng J. Lineage-specific exosomes promote the odontogenic differentiation of human dental pulp stem cells (DPSCs) through TGFβ1/smads signaling pathway via transfer of microRNAs. Stem Cell Res Ther. 2019 Jun 13;10(1):170. doi:10.1186/s13287-019-1278-x PubMed PMID: 31196201; PubMed Central PMCID: PMC6567518.

159.	Chiricosta L, Silvestro S, Gugliandolo A, Marconi GD, Pizzicannella J, Bramanti P, et al. Extracellular Vesicles of Human Periodontal Ligament Stem Cells Contain MicroRNAs Associated to Proto-Oncogenes: Implications in Cytokinesis. Front Genet. 2020;11:582. doi:10.3389/fgene.2020.00582 PubMed PMID: 32582296; PubMed Central PMCID: PMC7287171.

160.	Huang X, Deng Y, Xiao J, Wang H, Yang Q, Cao Z. Genetically engineered M2-like macrophage-derived exosomes for P. gingivalis-suppressed cementum regeneration: From mechanism to therapy. Bioact Mater. 2024 Feb;32:473–87. doi:10.1016/j.bioactmat.2023.10.009 PubMed PMID: 37965240; PubMed Central PMCID: PMC10640966.
